# Femtosecond Time- and Spectrally Resolved Ion Photofragmentation Spectroscopy: Case Studies of Two Alkylbenzene Cations

**DOI:** 10.1021/acs.jpca.5c04149

**Published:** 2025-07-21

**Authors:** Chen-Yi Chu, Hsin Liu, Po-Yuan Cheng

**Affiliations:** Department of Chemistry, 34881 National Tsing Hua University, Hsinchu 300044 Taiwan, Republic of China

## Abstract

Ultrafast photoionization-induced ionic relaxation dynamics in *n*-propylbenzene and 2,2-dimethylpropylbenzene cations were investigated using time- and spectrally resolved ion photofragmentation spectroscopy with a femtosecond photoionization–photofragmentation (PI–PF) detection scheme. Photoionization was initiated via 1 + 1 REMPI using femtosecond UV pump pulses well below the strong-field ionization regime, and the evolving ionic systems were probed by delayed visible-wavelength probe pulses to induce photofragmentation. Despite substantial ionic fragmentation induced by UV photoionization, the observed parent ion depletion transient signals can be attributed exclusively to the relaxation dynamics of intact parent cations generated at the two-photon level. Highly excited cationic states initially accessed by absorption of additional UV photons within the pump pulse do not contribute to the parent ion depletion transient signals, although their dynamics may appear in fragment ion formation transients. Ultrafast time-resolved photofragmentation spectra, obtained by measuring probe-induced parent ion depletion yield as a function of wavelength at a fixed delay time, reveal prominent ionic resonance absorption in the visible region. The observed time invariance of ion depletion signals across this spectral range indicates the absence of substantial postionization relaxation processes capable of altering the nature or resonance absorption characteristic of the cation. These findings establish alkylbenzene cations as ideal reference systems for benchmarking ultrafast dynamics in more complex ionic systems that do undergo substantial structural or electronic transformations following photoionization.

## Graphical abstract

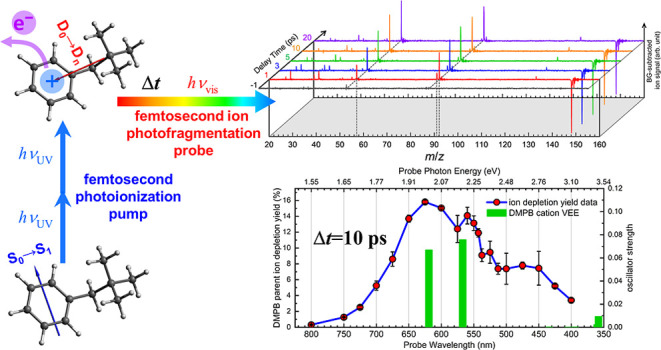

## 1 Introduction

Ionic reactions play a central role across a wide range of scientific fields and applications, ranging from astrochemistry^1^ and electrochemistry to biorelevant redox processes.^2^ In many of these systems, molecular ions are critically involved in mediating energy and charge transfer processes that govern key steps in complex mechanisms. Numerous spectroscopic techniques have been developed to elucidate the structures and dynamics of molecular ions in the gas phase.^3–10^ Over the past few decades, advances in ultrafast laser spectroscopy in the femtosecond^11–14^ to attosecond^15–17^ regimes have allowed investigations of ionic reactions with the temporal resolution necessary to capture nuclear and even electronic dynamics.

Our group has employed a variant of the conventional ultrafast pump–probe photoionization mass spectrometry that enables real-time investigations of ionic dynamics initiated by photoionization.^18–21^ This technique represents the ultrafast time-resolved implementation of ion photofragmentation spectroscopy, in which cations are first prepared by a femtosecond photoionization (PI) pump pulse and subsequently probed by photofragmentation (PF) induced by a delayed probe pulse. This approach, hereafter referred to as the PI–PF pump–probe scheme, is particularly suited for exploring ionic reactions, as removal of an electron from a neutral molecule can lead to substantial changes in nuclear and electronic structures. In well-designed molecular systems, such changes correspond to important elementary steps in chemistry, including isomerization, proton or charge transfer, and conformational relaxation. We have previously applied this femtosecond PI–PF technique to study unimolecular isomerization in azobenzene cation^18^ and intermolecular proton transfer reactions in phenol-ammonia^19,20^ and phenol-dimethylformamide^21^ complex cations, demonstrating its broad applicability to ultrafast ion chemistry.

In general, the ionization step can be initiated by femtosecond single-photon ionization, as well as by resonant^18–22^ and/or nonresonant multiphoton ionization (MPI).^23–26^ Other groups have employed strong-field ionization (SFI) using intense sub-50 fs laser pulses in the near-IR region.^27–43^ The ionization mechanisms under such conditions depend strongly on laser irradiance, among other crucial factors, such as the laser wavelength and the nature of molecular systems. However, it is generally accepted that when the laser irradiance is below 10^13^ W cm^–2^, photoionization proceeds predominantly via MPI.^44–48^ At laser irradiances between ∼10^13^ and 10^14^ W cm^–2^, tunnel ionization can occur,^34,49–51^ as the electric field of the radiation becomes comparable in strength to the binding force of valence electrons in molecules. In this regime, the Keldysh parameter (γ),^52^ originally formulated for atoms, serves as a rough boundary between MPI (γ≫1) and tunnel ionization (γ≪1) for polyatomic molecules.^50–52^

It is generally proposed that, in the tunnel ionization regime, only the most weakly bound electrons in the highest occupied molecular orbital (HOMO) respond to the quasi-static strong field, and ionization occurs adiabatically with minimal energy transfer to the molecular ion core. Many experimental studies have shown that SFI using intense femtosecond pulses indeed enhanced productions of intact molecular ions with suppressed fragmentation yields,^49,53–56^ except in cases where postionization ionic resonance absorption occurs.^28,34,44,57–59^ Such ionic absorption can be avoided by using nonresonant wavelengths in the near-IR region, allowing SFI to prepare ground-state molecular ions as a “launch state” for coherent control studies.^29^ On the other hand, there are theoretical^51,60–62^ and experimental studies^40–42,63–70^ indicating that SFI can also proceed via a nonadiabatic multielectron mechanism, in which inner valence electrons also respond to the strong filed, allowing access to multiple electronic continua corresponding to different molecular ionic valence orbitals.

At even higher laser irradiances above ∼10^14^ W cm^–2^, electron recollision driven by the reversed strong field, a phenomenon responsible for high-harmonic generation (HHG) and the generation of attosecond pulses, becomes possible.^35,71^ In this regime, the recollision process can produce ions with broader internal energy distributions, depending on the ponderomotive energy imposed by the oscillating strong field. The resulting ionic fragmentation patterns reflect the internal energy distributions and can serve as a guide for tailoring SFI conditions to mimic those encountered in electron ionization (EI) mass spectrometry.^35–39^

Thus, SFI can occur through multiple ionization pathways, and achieving a single dominant mechanism is often challenging. Moreover, it has been argued that reaching the tunnel ionization regime with multicycle pulses is practically difficult, particularly for molecules with relatively low ionization energies (<10 eV),^72^ because MPI can become substantial or even saturated on the rising edge of the pulse, before tunnel ionization regime is reached near the peak.^72^

In the present study, photoionization is initiated using one-color 1 + 1 resonance-enhanced multiphoton ionization (REMPI) with ∼120 fs UV laser pulses under conditions well-below the SFI regime. UV-REMPI offers the advantage of spectral selectivity, enabling specific molecular moieties to act as chromophores for ionization within larger molecular systems. Moreover, by accessing selected neutral excited states, it is possible to preferentially populate specific cationic excited states according to photoionization propensity rules. Notably, as demonstrated in this work, the observed parent ion depletion transient signals reflect exclusively the dynamics of intact parent cations produced at the two-photon level, despite the substantial ionic fragmentation induced by the UV pump pulse. Probing is achieved through resonance absorption of the evolving ionic system in the visible to near-IR region, which in most cases provides sufficient energy to induce ionic fragmentation.

These advantages have been exploited in our previous studies of photoionization-induced proton transfer in phenol–ammonia^19,20^ and phenol–dimethylformamide^21^ complexes, where the phenol S_1_ state was used as the chromophore to launch the nascent cation near the neutral nonproton-transferred geometry with the positive charge initially localized in the phenol moiety. The resulting ionic system then evolves toward the proton-transferred configuration. Proton-transfer dynamics was probed by monitoring the transformation of resonance absorption chromophores from the initial reactant (phenol cation) to the final product (phenoxy radical).

Recently, we have undertaken studies of intramolecular ion chemistry induced by femtosecond photoionization in a series of bifunctional molecules containing a phenyl ring and a functional group linked by an alkyl chain. By using the phenyl ring as a chromophore, these systems can be efficiently ionized via 1 + 1 REMPI through the neutral S_1_ state, allowing us to probe the ionic relaxation dynamics following photoionization. The interaction between the phenyl ring and the functional group changes markedly upon photoionization, driving the cation to undergo various relaxation processes depending on the nature of the system. However, to interpret the dynamical behaviors observed in such molecules, it is necessarily to first understand simpler model systems where substantial relaxation is not expected. To this end, we present case studies of two simple alkylbenzenes, *n*-propylbenzene (PB) and 2,2-dimethylpropylbenzene (DMPB) cations (see Scheme 1 for molecular structures), in which neither intramolecular ionic reactions nor substantial conformational changes are expected following photoionization. DMPB was specifically chosen because the presence of a *tert*-butyl group is expected to provide low-energy ionic dissociation pathways. The results of this study serve as a control system for interpreting experimental results from more complex systems that undergo ionic reactions or conformational changes after photoionization.

**Scheme 1:**
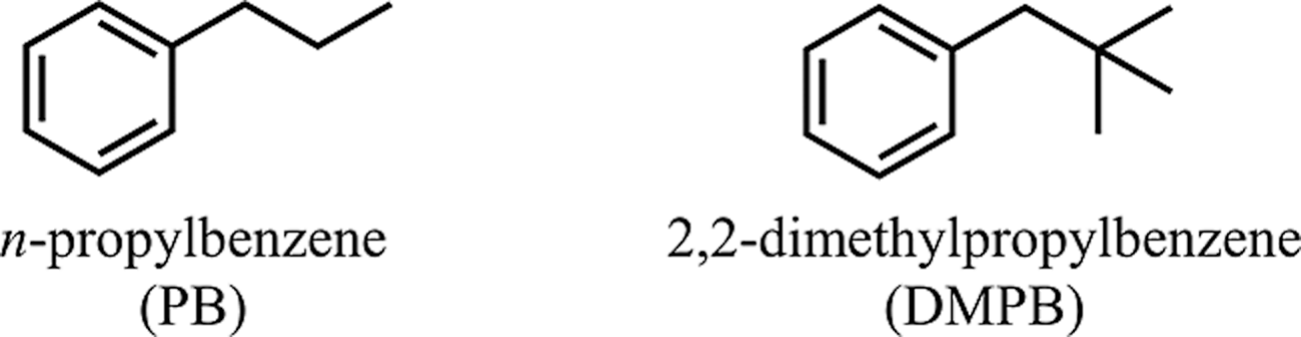
Molecular Structures of PB and DMPB

## 2 Methods

### 2.1 Overview and Experimental Considerations for Femtosecond PI–PF Spectroscopy

The femtosecond PI–PF pump–probe scheme described here can be regarded as an ultrafast time-domain analog of ion PF spectroscopy. In the conventional frequency-domain version, ions produced by photoionization or other means are typically cooled by supersonic expansion or thermal equilibration prior to probing in order to reduce spectral congestions.^3–10^ However, for the ultrafast time-domain version, postionization cooling is undesirable, as the subsequent ultrafast relaxation processes are the primary focus of investigation. Since the laser irradiances employed here are well below the SFI regime, the following description is framed within the context of the laser MPI-dissociation model.^45,46^

The basic principle of femtosecond PI–PF spectroscopy is schematically illustrated in Figure 1a, using typical alkylbenzene energetics as examples. A femtosecond UV pump pulse first produces an ensemble of parent molecular cations via 1 + 1 REMPI through the S_1_ state of the neutrals. For substituted benzenes, the S_1_ state typically lies slightly above half of the first ionization energy (IE) and has a lifetime much longer than the duration of the UV pump pulse used here. Therefore, femtosecond 1 + 1 REMPI can efficiently produce cations with initial structures closely resembling the neutral ground state. Depending on the nature of the system, the nascent cations may undergo relaxation dynamics that can be related to a wide range of elementary steps in chemistry. To probe the subsequent relaxation, a delayed femtosecond probe pulse in the visible to near-IR region is introduced to promote the evolving ionic system to excited states energetically capable of undergoing fragmentation. The dynamics of the initial cationic state can then be monitored by tracking either the parent ion depletion or the fragment ion formation as a function of the pump–probe delay time, provided that the probing transition is sensitive to the evolving ionic structure or electronic configuration.

**Figure 1:**
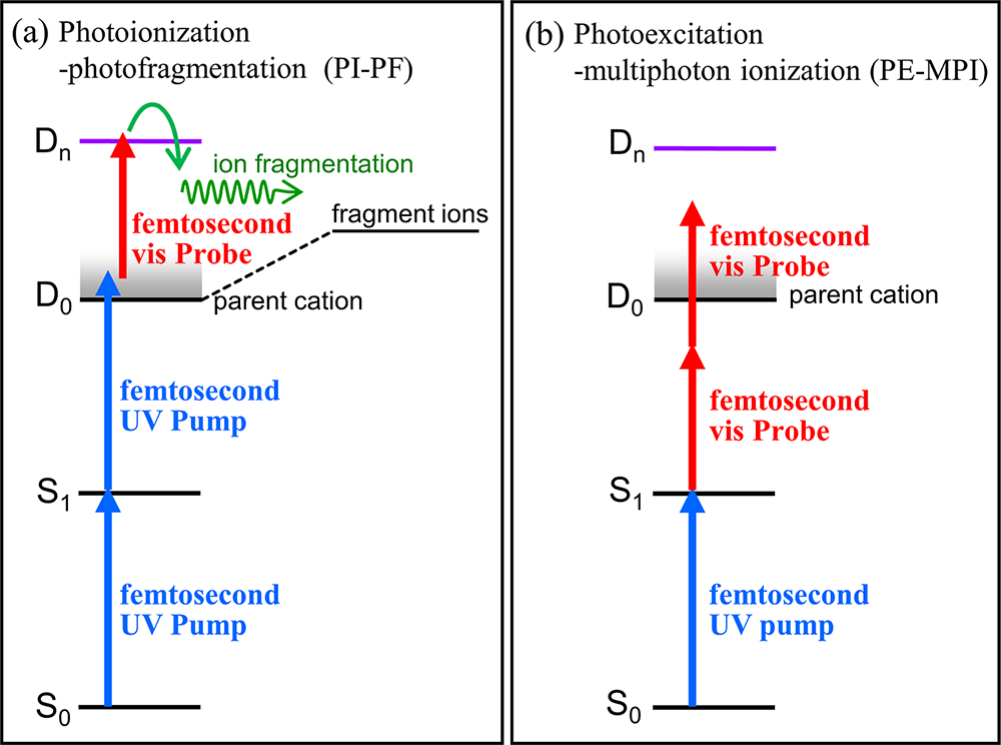
Schematic illustrations of two parallel pump–probe detection schemes discussed in the text: (a) photoionization–photofragmentation (PI–PF), and (b) photoexcitation-multiphoton ionization (PE–MPI) schemes. Relevant energy levels are drawn approximately to scale, reflecting typical alkylbenzene energetics. Blue and red arrows represent the UV pump and visible-region probe photons, respectively. Green curved and wavy arrows denote nonadiabatic and/or adiabatic pathways leading to fragmentation.

The scheme described above is employed in the present study for alkylbenzenes. However, the initial cation preparation can, in general, be achieved through alternative REMPI pathways involving other intermediate states or even via nonresonant MPI.^23–26^ Subsequent ion fragmentation following the probe transition depends on the nature of the accessed ionic excited states and may proceed via multiple pathways. Common mechanisms include adiabatic dissociation on excited-state potential energy surfaces or internal conversion to the cationic ground state followed by slower unimolecular dissociations.

Although the femtosecond PI–PF scheme outlined above is conceptually straightforward for exploring ionic dynamics, its experimental arrangement is practically identical to that of the conventional ultrafast photoexcitation–multiphoton ionization (PE–MPI) scheme commonly used to study neutral excited-states dynamics, as shown in Figure 1b. Because both schemes can occur in parallel and produce indistinguishable ion signals, cares must be taken to minimize PE–MPI contributions to avoid obscuring the ionic dynamics in the measured transients. For typical benzene derivatives, one can take advantage of the fact that their S_1_ states generally require absorption of at least two vis/near-IR probe photons to reach the ionization threshold, while dissociation of their cation ground states typically require only a single probe photon. Therefore, a weak probe pulse should be used to suppress two-photon ionization of the neutral S_1_ state and minimize PE-MPI contributions, while maintaining sufficient one-photon dissociation efficiency for the cation.

On the other hand, when probing neutral S_1_-state dynamics using the PE–MPI scheme, the pump irradiance must be reduced to a low level at which the pump pulse generates essentially no detectable cation signal; otherwise, contributions from ionic dynamics may contaminate the measured ionization transients. In this case, a stronger probe pulse is usually required to facilitate MPI of the neutral S_1_ state. Thus, the dominance of either the PI–PF or PE–MPI scheme can be controlled by adjusting the relative irradiances of the pump and probe pulses.

### 2.2 Experimental Section

The experimental setup is similar to those described in our previous reports;^18^ therefore, only key features and procedures specific to the present study are summarized here. Briefly, the outputs of a 1 kHz amplified femtosecond Ti:sapphire laser system (Spectra Physics, Tsunami and Spitfire) and a traveling-wave optical parametric amplifier (Light Conversion, TOPAS) were used to produce pump pulses at 266.4 nm and probe pulses in the 400–800 nm spectral range via various nonlinear optical conversion processes.

The experiments were conducted in a two-chamber, differentially pumped molecular beam apparatus equipped with a time-of-flight mass spectrometer (TOF-MS). A gas mixture, prepared by flowing pure He gas through a cold trap containing liquid PB or DMPB cooled to about −10 °C, was expanded at a backing pressure of ∼300 Torr through a 100 μm diameter pinhole to produce a continuous supersonic jet in the first chamber. The jet was skimmed before entering the second chamber, where it was intersected by the femtosecond laser pulses in the extraction region of the TOF-MS. The probe beam was directed through a computer-controlled optical delay line and focused collinearly with the pump beam using a *f* = 300 mm CaF_2_ lens into the molecular beam. Unless otherwise indicated, the relative polarizations of the pump and probe beams were set at the magic angle (54.7°).

Mass-selected transients were recorded by monitoring the ion signal at the mass channel of interest using a boxcar integrator (Stanford Research SR250) while the pump–probe delay time was scanned. The effective instrument response functions (IRF) of the pump and probe pulses were measured in situ using the nonresonant pump–probe MPI signal of Xe atom. The full width at half-maximum (fwhm) of the IRF ranged from 140 to 200 fs, depending on the probe wavelength. The IRF trace was also used to define time zero for pump–probe measurements.

The procedure for measuring parent ion depletion yields is described in Section 3.2. Consistent and optimized spatial overlap of the pump and probe pulses was ensured by adjusting steering mirrors to maximize the nonresonant MPI signal of Xe atom at time zero prior to each measurement. The focal volume of the probe beam across the 400–800 nm wavelength range was optimally matched to that of the pump by adjusting a telescope placed upstream in the probe beam path.

Laser pulse energies were controlled using variable neutral density filters placed in laser beam paths. Unless otherwise noted, PI–PF measurements were performed using a UV pump pulse energy (ε_pump_) of ∼5.8 μJ/pulse, corresponding to an estimated irradiances of ∼2.3 × 10^12^ W cm^–2^.^73^ The probe pulse energy (ε_probe_) was varied between ∼0.4 and ∼1.4 μJ/pulse, depending on the specific measurement requirements. In the following discussion, pulse energies are reported rather than irradiances, as the former were directly measured using a calibrated energy meter, while the latter were estimated from the measured pulse energies and other laser beam characteristics.

## 3 Results and Analyses

The goal of this study is to investigate the ultrafast dynamics of PB and DMPB cations produced via femtosecond 1 + 1 REMPI at 266.4 nm through their neutral S_1_ states. Nanosecond 1 + 1 REMPI spectra of PB and DMPB have been reported previously.^74–76^ For PB, two S_1_-state origins were observed at 266.43 and 266.08 nm, corresponding to the *anti* and *gauche* conformers, respectively.^74,75^ For DMPB, only a single conformer with its S_1_-state origin at 266.43 nm was identified.^76^ Consequently, given the coherent bandwidth of ∼0.85 nm (∼120 cm^–1^) associated with the ∼120 fs pump pulse at 266.4 nm, the UV pump pulse produces cations by first resonantly exciting both molecules through their respective S_1_-state origins, followed by photoionization transitions.

### 3.1 Femtosecond UV Photoionization Mass Spectra

The initial ionic ensemble produced by the femtosecond UV (∼266.4 nm) pump pulse can be partially characterized by examining the laser irradiance dependence of the resulting mass spectra. Figure 2a,b show TOF mass spectra of PB and DMPB, respectively, photoionized with six representative pulse energies for comparison. At the highest UV pulse energy used here (∼10 μJ/pulse; ∼4 × 10^12^ W cm^–2^),^73^ the Keldysh parameter (γ) is about 12.7 for both alkylbenzenes with IEs of ∼8.7 eV,^74,75,77^ indicating that the photoionization conditions employed here are well below the SFI regime. Accordingly, the observed laser irradiance dependence of mass spectra can be interpreted within the framework of the MPI-dissociation model.^45,46^

**Figure 2:**
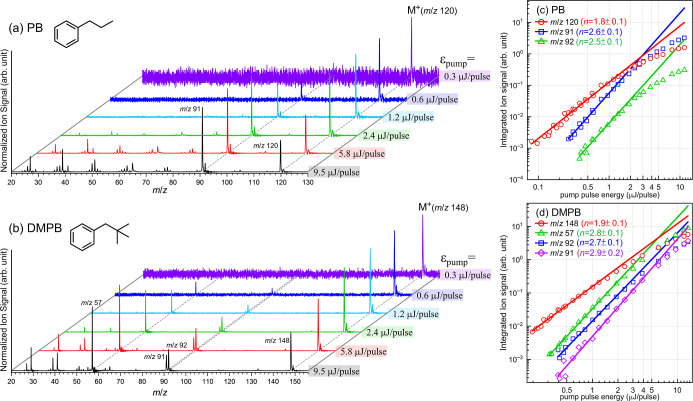
TOF mass spectra of (a) PB and (b) DMPB photoionized by the femtosecond UV (266.4 nm) pump pulse alone at six representative pulse energies (ε_pump_), as indicated. Each spectrum is normalized to its most intense peak and offset for clarity and ease of comparison. (c,d) Log–log plots of the integrated ion signal versus UV laser pulse energy, derived from TOF mass spectra of PB and DMPB, respectively, photoionized using UV pulse energies ranging from 0.1 to 14 μJ/pulse. Straight lines are the results of simple linear regression applied to data points within the linear region of the log–log plots. The slope (*n*) of each best-fit line is given for the corresponding ion in each plot.

At extremely low pulse energies (<0.3 μJ/pulse), only the parent molecular ions of PB^+^ (*m*/*z* 120) and DMPB^+^ (*m*/*z* 148) are observed. As the pulse energy increases (>0.5 μJ/pulse), several major fragment ions begin to appear. For DMPB, the most abundant fragment ion is observed at *m*/*z* 57, corresponding to the formation of a *tert*-butyl cation (C­(CH_3_)_3_^+^) and a benzyl radical (PhCH_2_^•^). Both species are relatively stable, leading to a low-energy dissociation pathway for DMPB^+^ (see Table 1).^78,79^ The next most abundant fragment ions, *m*/*z* 91 (C_7_H_7_^+^) and *m*/*z* 92 (C_7_H_8_^+^), are commonly observed in EI mass spectra of alkylbenzenes.^80^ The *m*/*z* 91 ion signal likely arises from a combined contribution of benzylium and tropylium cations. The *m*/*z* 92 ion is believed to originate from a γ-hydrogen rearrangement,^81–83^ a variant of the well-known McLafferty rearrangement, which is favored in DMPB^+^ due to the abundance of γ-hydrogen atoms in the *tert*-butyl group. Formation of *m*/*z* 92 (C_7_H_8_^+^) ions with structures different from the methylene-2,4-cyclohexadiene (MCD) cation, such as the toluene cation, involve higher barriers, as suggested by previous studies.^83^

**Table 1:** Enthalpies of Reaction Derived from Available Thermochemical Data for the Production of Major Fragment Ions from the PB and DMPB Cations^a^

	Δ_r_ *H*_298 K_ ^a^	
cationic dissociation reaction	kJ/mol	eV
(PB^+^)^b^→ C_7_H_7_^+^(*m*/*z* 91, benzylium cation)^c^+ C_2_H_5_^•^ (ethyl radical)^c^	182	1.89
(PB^+^)^b^→ C_7_H_7_^+^(*m*/*z* 91, tropylium cation)^c^+ C_2_H_5_^•^ (ethyl radical)^c^	151	1.56
(DMPB^+^)^b^→ (CH_3_)_3_C^+^(*m*/*z* 57, *tert*-butyl cation)^c^+ C_6_H_5_CH_2_^•^ (benzyl radical)^c^	138	1.43
(DMPB^+^)^b^→ C_7_H_7_^+^(*m*/*z* 91, benzylium cation)^c^+ (CH_3_)_3_C^•^ ^c^	176	1.83
(DMPB^+^)^b^→ C_7_H_7_^+^(*m*/*z* 91, tropylium cation)^c^+ (CH_3_)_3_C^•^ ^c^	146	1.51
(DMPB^+^)^b^→ C_7_H_8_^+^(*m*/*z* 92, MCD^e^cation)^d^+ CH_2_C(CH_3_)_2_ (isobutene)^c^	133	1.38

a Enthalpies of reaction were calculated from the heats of formation of the reactant and product species based on available data.

b From ref 78.

c From ref 79.

d From ref 83.

e MCD = methylene-2,4-cyclohexadiene.

For PB, the most intense fragment ion appears at *m*/*z* 91, whereas the *m*/*z* 92 signal is slightly greater than the expected natural abundance of the ^13^C peak for C_7_H_7_^+^ by only ∼2% even at the highest pulse energy used here.^84^ This indicates that dissociation pathways leading to the production of *m*/*z* 92 (C_7_H_8_^+^) are not active, although a few channels with low dissociation limits exist. A previous study of photodissociation of PB^+^ in the near-UV and visible spectral regions also reported negligible production of the *m*/*z* 92 (C_7_H_8_^+^) fragment ion.^85^

These observations clearly indicate that the parent and fragment ions exhibit different laser irradiance dependences. Quantitative analyses of the laser pulse energy dependence of the parent and major fragment ion yields, shown in Figure 2c,d, suggest that both PB and DMPB parent cations are produced via absorption of two UV photons, whereas the production of major fragment ion requires absorption of at least three UV photons. This is consistent with the fact that the total energy of two 266.4 nm photons (∼9.3 eV) exceeds the adiabatic IEs of both PB and DMPB (∼8.7 eV)^74,75,77^ but remains below the dissociation limits for all major fragment ions (see Table 1).

Major fragment ions are most likely produced via absorption of a third UV photon by the nascent cation, following the initial 1 + 1 REMPI process. This occurs through the molecular-ion “ladder climbing” mechanism^44–48^ within the UV pump pulse to access cationic excited states with energies (∼4.65 eV) that are sufficient to undergo ionic dissociation (see Table 1). The ladder climbing mechanism can become efficient when the pump wavelength is resonant with cationic excited states. As discussed below, the high density of electronic states in alkylbenzene cations in the UV region makes such scenario unavoidable, even at moderate pump irradiances.

At even higher pulse energies (>2 μJ), fragment ions with lower masses become non-negligible and clearly exhibit irradiance dependences distinct from the heavier major fragment ions, indicating that their formation requires absorption of more than three UV photons. Most of these lighter fragment ions are also observed in their respective EI mass spectra.^80,86^

Consequently, depending on the UV pulse energy used, the initial parent ion ensembles are produced with a wide range of internal energies, reflecting different orders of multiphoton excitation. Hereafter, cations formed via absorption of two UV photons are referred to as being produced at the 2*h*ν_uv_ level, while those formed via absorption of a third UV photon within the pump pulse are referred to as being produced at the 3*h*ν_uv_ level. Since photoionization produces a distribution of vibrational energies in the cationic ground state, the 2*h*ν_uv_ level, as defined here, corresponds to an energy range between the adiabatic IE and the total energy of two UV photons, i.e., ∼8.7–9.3 eV. Similarly, the 3*h*ν_uv_ level spans an energy range of ∼13.35–13.95 eV.

In the following time-resolved experiments, a pulse energy of ∼5.8 μJ (∼2.3 × 10^12^ W cm^–2^)^73^ was used for the 266.4 nm UV pump pulse, as a sufficiently strong parent ion signal was essential to achieve reasonable signal-to-noise (S/N) ratios in the ion depletion measurements. Under this condition, substantial parent and major fragment ions are present in the mass spectra, as shown in Figure 2, indicating that while a significant fraction of ions is generated at the 2*h*ν_uv_ level, a notable portion of the initial cations is produced at the 3*h*ν_uv_ level. The latter can undergo efficient fragmentation, resulting in the formation of major fragment ions at the expense of the parent ion population. In the following discussion, these mass spectra are referred to as the background, representing ion signals generated by the UV pump pulse alone in the absence of the probe pulse.

### 3.2 Observation and Measurement of Parent Ion Depletion

Under typical conditions described in Section 2.2 for PI–PF measurements, parent ion depletion transients were readily observed. Figure 3a, trace (i), shows a typical femtosecond PI–PF transient of the PB parent cation measured at λ_probe_ = 500 nm, while trace (iv) displays the baseline signal, recorded with both lasers blocked under otherwise identical conditions, reflecting the intrinsic offset of the boxcar integrator. Clearly, the pump–probe transient exhibits a pronounced and sudden decrease in the PB parent ion signal near time zero, followed by a constant depleted ion signal. This transient was recorded under laser conditions optimized for the PI–PF scheme, using a relatively intense pump pulse (ε_pump_ ∼ 5.8 μJ/pulse) to produce a large PB^+^ population via 1 + 1 REMPI, and a much weaker probe pulses (ε_probe_ ∼ 0.7 μJ/pulse) to dissociate the evolving ionic system. Background signals recorded under identical conditions but with either the pump or probe laser blocked are shown in traces (ii) and (iii), respectively. The pump-only condition trace (ii) yields a large PB^+^ ion signal that matches the negative-time signal in trace (i), while the probe-only condition (trace (iii) produces no detectable PB^+^ ion signal, as evidenced by its closeness to the baseline trace (iv). The sum of traces (ii) and (iii), after baseline subtraction, matches the signal in the negative-time region of trace (i), confirming that the signal in this region represents the time-independent background ion signal produced solely by the pump pulse. Similar results are also observed for the DMPB cation measured at λ_probe_ = 650 nm, as shown in Figure 3b, with an even more pronounced depletion signal. These observations confirm that the decrease of the ion signal in the positive-time region arises from the depletion of the PB and DMPB parent ion populations when the probe pulse arrives after the pump.

**Figure 3:**
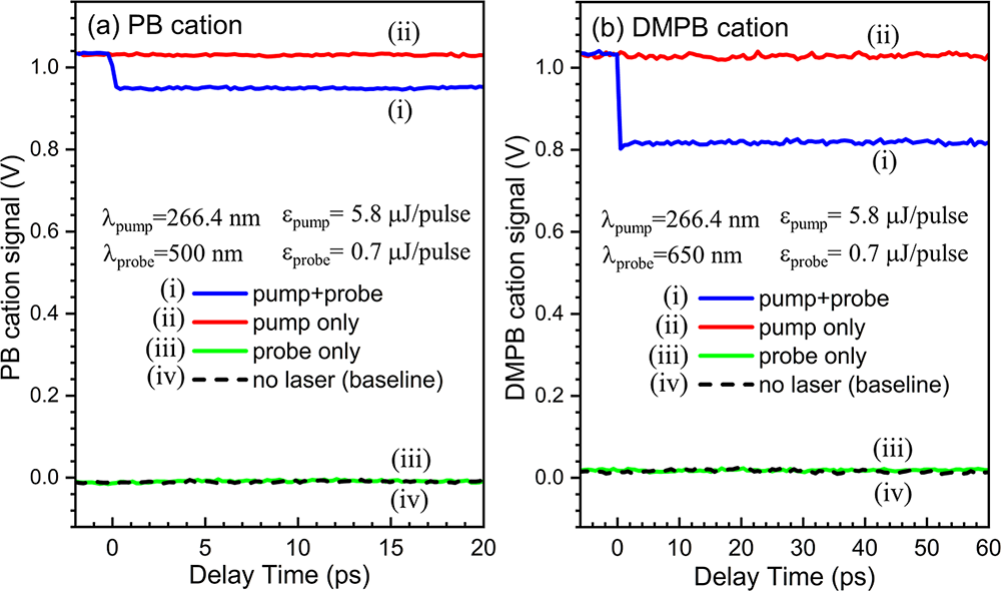
Manifestation of parent ion depletion transient signals for (a) PB^+^ at λ_probe_ = 500 nm and (b) DMPB^+^ at λ_probe_ = 650 nm under typical PI–PF detection conditions, using a UV pump pulse energy of 5.8 μJ/pulse and a probe pulse energy of ∼0.7 μJ/pulse. Trace (i): pump–probe parent ion transient under PI–PF detection conditions. Trace (ii): parent ion signal with the pump laser only. Trace (iii): parent ion signal with the probe laser only. Trace (iv): baseline signal recorded with both pump and probe lasers blocked. Note that the nonzero baseline signal arises from the intrinsic offset of the boxcar integrator.

The time-dependent parent ion depletion yield, Φ­(*t*), is defined as

1 $$\Phi \left(t\right)=\left(\frac{S_{bg}-S\left(t\right)}{S_{bg}}\right)\times 100\%$$

where *S*_bg_ is the average time-independent parent ion background signal in the negative-time region, and *S*(*t*) is the time-dependent parent ion signal at pump–probe delay time *t*. Both *S*_bg_ and *S*(*t*) can be obtained from typical scans, such as those shown in Figure 3 (trace (i)), after subtracting the baseline signal (trace (iv)). To avoid long-time signal drift, the integrated ion signal was measured at only a few selected positive and negative delay times and rapidly scanned back and forth, accumulating ∼6 × 10^4^–2.4 × 10^5^ laser shots average over a short period of time (∼1.5–6 min). The average baseline signal was measured immediately before and after each pump–probe scan in the same manner with both lasers blocked, and this was subtracted from the pump–probe signal to determine *S*_bg_(*t*) and *S*(*t*) for use in eq 1.

Figure 4a,b show log–log plots for the measured parent ion depletion yield as a function of probe laser pulse energy for PB and DMPB cations, respectively, at a fixed delay time of ∼10 ps. In both cases, the parent ion depletion yield exhibits a linear dependence on pulse energy below ∼1 μJ/pulse, indicating that the ion depletion arises from one-photon absorption of the probe pulse followed by fragmentation. At pulse energies above ∼1 μJ/pulse, saturation behavior gradually becomes evident, primarily due to absorption saturation and, to a lesser extent, increased two-photon ionization of the neutral S_1_ state, i.e., the PE–MPI mechanism, which enhances the parent ion signal and thereby reduces the observed depletion yield. To avoid interference from such saturation effects while maintaining reasonably high depletion yields, probe pulse energies used in parent ion depletion measurements in this study were kept below or near the saturation threshold.

**Figure 4:**
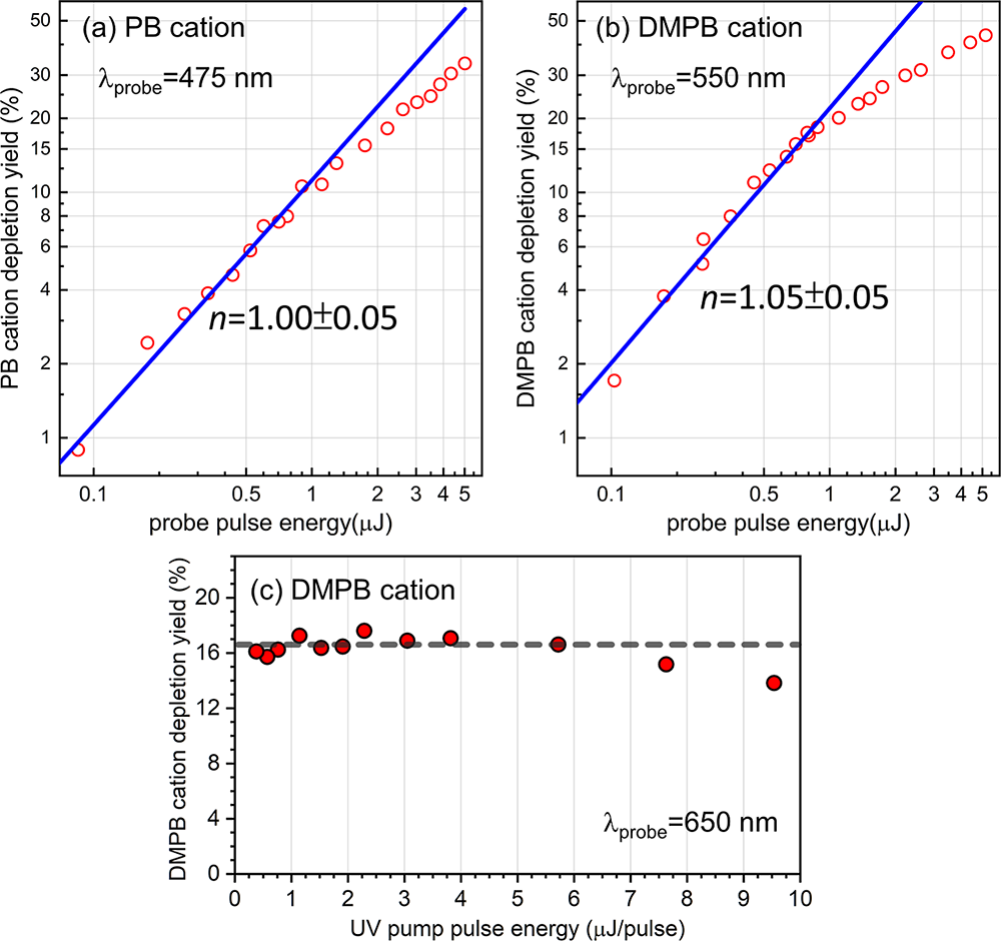
Log–log plots of the parent ion depletion yield verse probe laser pulse energy for (a) PB^+^ and (b) DMPB^+^, with the UV pump pulse energy held constant at 5.8 μJ/pulse. The straight lines are the results of simple linear regression fits to the data points in the roughly linear region, and the slopes (*n*) of the best-fit lines are given in each panel. (c) Pump pulse energy dependence of DMPB parent ion depletion yields, measured with the probe pulse energy held constant at ∼0.5 μJ/pulse while the UV pump pulse energy was varied from ∼0.3 to ∼10 μJ/pulse. The horizontal gray dashed line is drawn as a visual guide.

In contrast to the nearly linear dependence on probe pulse energy, the parent ion depletion yield shows no significant dependence on pump pulse energy, except when the latter is reduced to very low levels below ∼0.3 μJ/pulse. As shown in Figure 4c, the DMPB parent ion depletion yield remains nearly constant over the range of ∼0.3–6 μJ/pulse. At higher pump pulse energies, a slight decrease in depletion yield is observed, likely due to an increased fraction of ions generated outside the spatial overlap of the pump and probe focal volumes.

### 3.3 Pump–Probe Polarization Dependence

Figure 5 displays PI–PF parent ion depletion transients of PB^+^ and DMPB^+^ measured at 475 and 650 nm, respectively, at three relative pump–probe polarization angles: 0° (parallel), 54.7° (magic angle), and 90° (perpendicular), under otherwise identical conditions. Clearly, these alkylbenzene parent ion depletion transients exhibit strong dependence on relative pump–probe polarization. The distinct temporal behaviors observed between the parallel and perpendicular relative polarizations within the first few picoseconds are characteristic of rotational coherence dephasing. In contrast, transients recorded at the magic angle eliminate the influence of rotational coherence, yielding transients that reflect the intrinsic dynamics of the ionic system.

**Figure 5:**
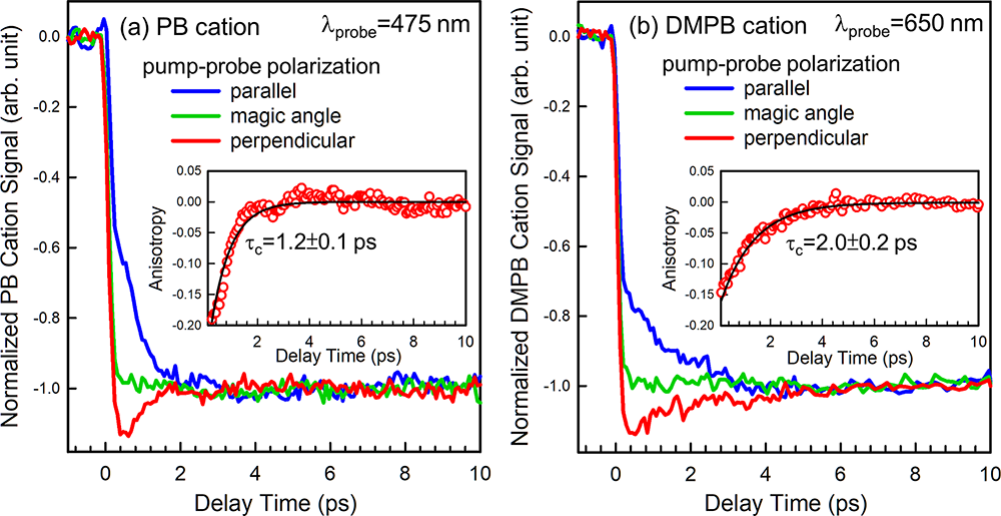
Parent ion depletion transients of (a) PB^+^ and (b) DMPB^+^ measured at three relative pump–probe polarization angles. Blue traces: 0° (parallel); green traces: 54.7° (magic angle); and red trace: 90° (perpendicular). PB^+^ transients were measured at λ_probe_ = 475 nm, and DMPB^+^ transients at λ_probe_ = 650 nm. These transients were background-subtracted and normalized to the same asymptotic signal at ∼10 ps. The time-dependent anisotropy, calculated from the parallel and perpendicular data using eq 2, is shown in the insets. The solid black lines are single-exponential rise fits to the anisotropy data (open circles).

These results highlight the importance of measuring ion depletion transients with the pump–probe polarization set at the magic angle. Rotational coherence dynamics has been extensively studied, primarily for neutral excited states, using various ultrafast pump–probe spectroscopic techniques.^87–90^ In the present case, rotational alignment in the cation is initiated by 1 + 1 REMPI via the S_1_ state to the ionization continuum. The pronounced polarization dependence observed here indicates that the 1 + 1 REMPI process preserves the initial rotational coherence during cation formation.

The time-dependent anisotropy, *R*(*t*), of the ion depletion signal can be evaluated by

2 $$R\left(t\right)=\frac{\Phi_{∥}\left(t\right)-\Phi_{⊥}\left(t\right)}{\Phi_{∥}\left(t\right)+2\Phi_{⊥}\left(t\right)}=\frac{S_{∥}^{\prime }\left(t\right)-S_{⊥}^{\prime }\left(t\right)}{S_{∥}^{\prime }\left(t\right)+2S_{⊥}^{\prime }\left(t\right)}$$

where Φ_∥_(*t*) and Φ_⊥_(*t*) are the parent ion depletion yields, as defined in eq 1, measured with the parallel and perpendicular pump–probe polarizations, respectively.^88,89^ As shown in eq 2, *R*(*t*) can equivalently be calculated using the background-subtracted parent ion signal, *S*′(*t*) = *S*(*t*) – *S*_bg_, shown in Figure 5. *R*(*t*) traces derived from *S*′(*t*) data are given in the insets. Note that the *S*′(*t*)­signals displayed here are normalized such that *R*(*t*) approaches zero at long delay times (∼10 ps), although under isolated condition a small residual anisotropy, comparable to the noise level, may be present.^87,90^

The polarization dependences of the parent ion depletion transients observed in Figure 5 are consistent with an orthogonal relative orientation between the pump and probe transitions. In fact, the initial anisotropies near time zero are close to the theoretical value of −0.2 for the case of orthogonal transition dipoles.^87–89^ In this study, parent ions are produced via 1 + 1 REMPI through the S_1_ states of alkylbenzenes. In the absence of photoelectron angular discrimination, ionization from the S_1_ state to the continuum is at most weakly polarized, and the overall dipole direction of the pump transition is therefore governed by the S_0_ → S_1_ excitation.

Under the C_2v_ point group approximation for monosubstituted alkylbenzenes, the S_1_ state typically has B_2_ symmetry, and the S_0_(^1^A_1_) → S_1_(^1^B_2_) transition dipole lies in the phenyl plane, oriented perpendicular to the phenyl–alkyl bond. Visible absorptions of their cation ground states (D_0_) are less well characterized, but are likely analogous to the dipole-allowed D_0_(^2^B_1_) → ^2^B_1_ transition that has been identified in some halogenated benzene cations.^91,92^ Therefore, under the C_2 V_ approximation, the visible resonance absorption of alkylbenzene cations is likely polarized along the phenyl-alkyl bond. As such, the pump and probe transitions are expected to be orthogonal, consistent with the observed polarization dependence. This orthogonality is further supported by our quantum chemical calculations presented below.

As shown in the insets of Figure 5, the time-dependent anisotropy decays smoothly to near zero, with DMPB^+^ exhibiting slower rotational dephasing than PB^+^. The coherence times, defined as the time required for the anisotropy to decay to one-fourth of the initial value,^89^ are ∼2 ps for DMPB^+^ and ∼1.2 ps for PB^+^. It has been shown that the thermally averaged rotational coherence time is proportional to (*TB*)^−1/2^,^89^ where *T* is the rotational temperature and *B* is the rotational constant of a symmetric-top molecule about either of its two identical principal axes. Thus, the longer rotational coherence time observed for DMPB^+^ is consistent with its smaller rotational constant, which results from the presence of a bulky *tert*-butyl group. Using rotational constants obtained from density functional theory (DFT) calculations presented below, the measured rotational coherence times give estimated rotational temperatures of ∼100–180 K, consistent with the soft expansion condition employed here.

Although relative polarization dependence provides insight into the orientation of the transition dipole moments involved, rotational coherence dynamics on the picosecond time scale complicate the interpretation of the ultrafast intrinsic ionic dynamics. Therefore, to minimize the influence of rotational coherence, all time-resolved measurements in this study, except those presented in this section, were conducted with the pump–probe polarization set at the magic angle.

### 3.4 Parent Ion Depletion Transients

Figure 6a,b display femtosecond PI–PF parent ion depletion transients for PB^+^ and DMPB^+^, respectively, measured at various probe wavelengths. In all cases, the transients exhibit a nearly instantaneous decrease in parent ion signal near time zero, followed by a constant signal level that persists up to the maximum delay time (∼1 ns) limited by the delay stage. The absence of noticeable temporal variation suggests that the subsequent relaxation dynamics of both alkylbenzene cations following 1 + 1 REMPI do not substantially affect the ion photofragmentation yield.

**Figure 6:**
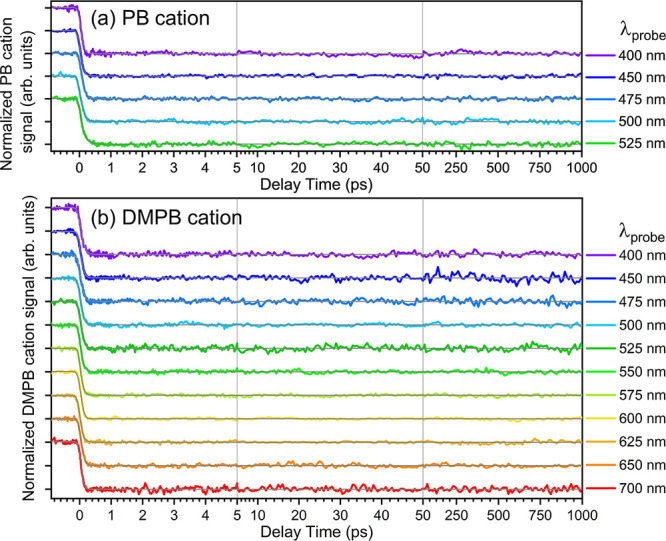
Parent ion depletion transients for (a) PB^+^ and (b) DMPB^+^ under PI–PF detection conditions, measured at various probe wavelengths. The UV (266.4 nm) pump pulse energy was fixed at 5.8 μJ/pulse, while the probe pulse energies were maintained below saturation, ranging from ∼0.72 to 1.4 μJ/pulse, depending on the probe wavelength. All transients shown in this figure are normalized to the same scale and vertically offset for clarity. Two breaks are included on the delay time axis at 5 and 50 ps to highlight temporal behaviors across three distinct time scales.

These ion transients were fitted using a model function, *S*(*t*) = *S*_bg_ – *M*(*t*), convoluted with a Gaussian IRF. *S*_bg_ represents the time-independent background parent ion signal produced by the pump pulse, and *M*(t) describes the time-dependent molecular response function of the evolving ionic system. In this study, *M*(t) is modeled as a step-like function with an initial ultrafast rise to account for early time behavior (see below). The results of the best fits are shown as gray solid lines in Figure 6a,b.

Early time behaviors of the parent ion depletion transients measured at several representative probe wavelengths are displayed in Figure 7, along with in situ IRF traces recorded at each wavelength using the nonresonant pump–probe MPI signal of Xe atoms. Early time transients recorded at other probe wavelengths are given in Figure S1. In all transients presented here, the depletion signal reaches its half-maximum level at positive delay times of several tens to ∼100 fs, indicating that the depletion signal does not appear instantaneously. This behavior persists in transients recorded with reduced pump and/or probe pulse energies (see Figure S2), suggesting that the delayed onset is not caused by saturation effects or nonlinear enhancement of ionization at time zero. As rationalized in Section 4.4, these early time transients can be described by a composite model consisting of an instantaneous rise, i.e., a step function, and an exponential rise with a time constant τ_1_. Here, the term “rise” refers to the *M*(t) function defined above and denotes an increase in depletion signal, which corresponds to a decrease in the observed ion signal. The rise time constants obtained from the best fits range from ∼60–110 fs for PB^+^ and ∼50–80 fs for DMPB^+^, indicating that the underlying dynamics is slightly faster in DMPB^+^.

**Figure 7:**
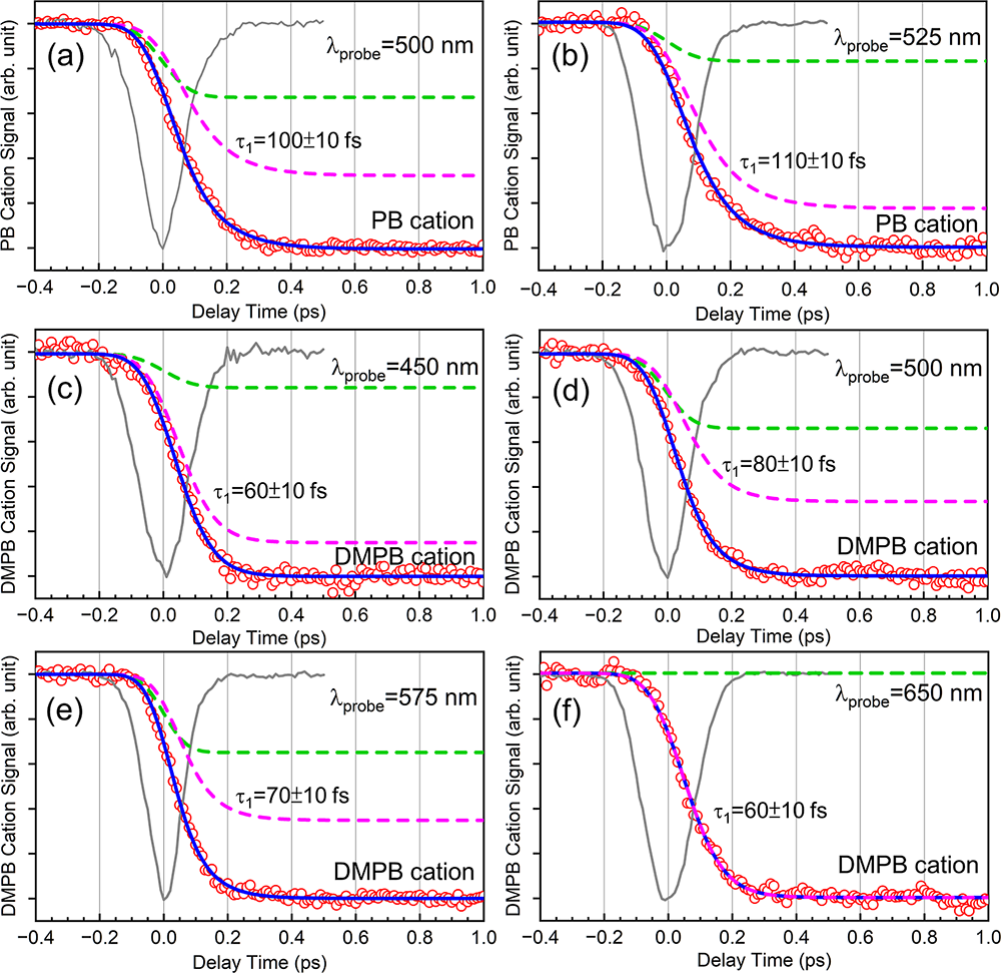
Early time parent ion depletion transients of (a,b) PB^+^ and (c–f) DMPB^+^ measured at selected probe wavelengths, shown with the best fits using the two-component model function described in the text. Inverted IRF traces recorded in situ at each probe wavelength are overlaid as gray lines. Red open circles are the experimental data, and blue solid lines are the best-fit curves. Colored dashed lines represent the decomposed components of the fit: (green) an instantaneous rise for parent cations with low vibrational excitation, and (pink) an exponential rise for parent cations with high vibrational excitation. Extracted rise times (τ_1_) from the best fits are also shown for each case.

The parent ion depletion transients reported here were measured with laser conditions optimized for the PI–PF detection scheme. The competing PE–MPI scheme, as described in Section 2.1, becomes important only when the probe pulse energy is raised to a much higher level. PE–MPI transients measured at λ_probe_ = 475 and 500 nm, using a reduced pump pulse energy (<0.05 μJ/pulse) and an elevated probe pulse energy (∼5–6 μJ/pulse), are given in Figure S3. Under these conditions, the transient signals become positive-going and remain constant after time zero. These parent ion “enhancement” transients result from two-photon ionization of the neutral S_1_ state by the probe pulse and reflect the neutral S_1_-state lifetimes, which are known to be at least several tens of nanoseconds near the S_1_-state origin for alkylbenzenes. The contribution of PE–MPI is expected to be negligible in the PI–PF transients presented in this study due to the much lower probe pulse energies employed.

### 3.5 Time-Resolved Ion Photofragmentation Mass Spectra

Figure 8a,b display background-subtracted time-resolved ion photofragmentation mass spectra (TRMS) of PB^+^ and DMPB^+^, respectively, measured at several pump–probe delay times with λ_probe_ = 500 nm. At positive delay times, the parent ion exhibits negative-going signals, indicating depletion of the parent ion population following the arrival of the probe pulse. In contrast, the fragment ion signals are positive-going, reflecting formation of fragment ions after the arrival of the probe pulse. The integrated intensity of the parent ion depletion closely matches the sum of integrated intensities of all fragment ions at each delay time,^93^ confirming that the fragment ions observed in these TRMS arise from probe-induced photodissociation of the parent ion.

**Figure 8:**
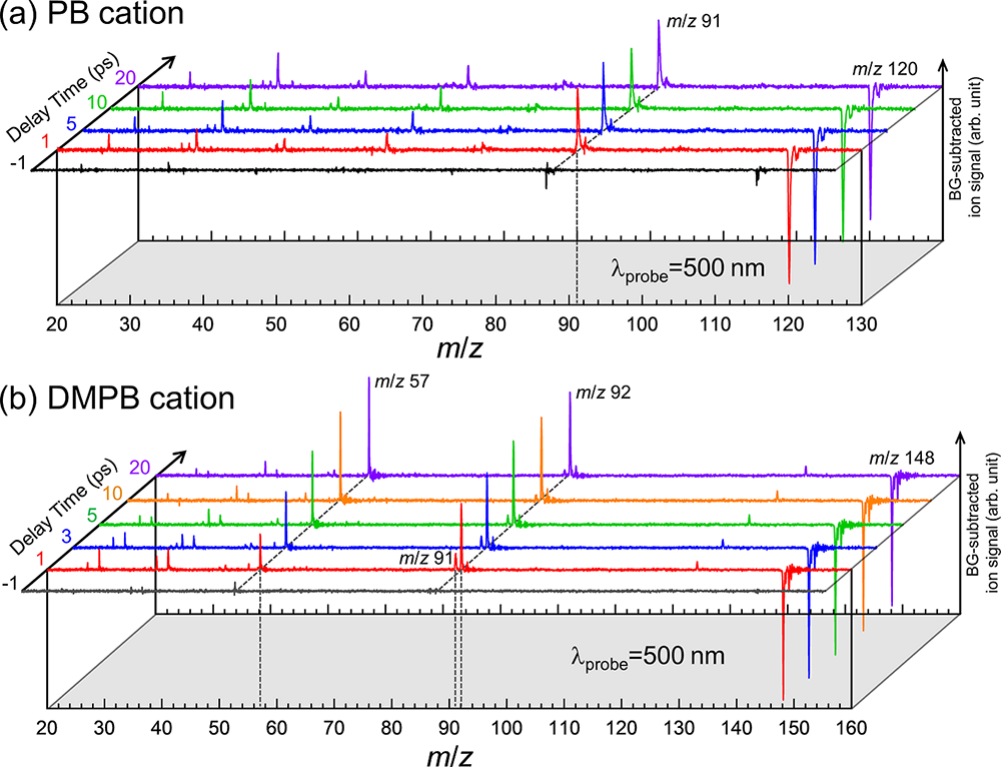
Background-subtracted time-resolved mass spectra of (a) PB^+^ and (b) DMPB^+^ measured at several representative pump–probe delay times under typical PI–PF conditions (ε_pump_ = 5.8 μJ/pulse, ε_probe_ = 1.1 μJ/pulse) at λ_probe_ = 500 nm. Each trace was obtained by subtracting the background mass spectrum recorded at a negative delay time (<−1 ps) from that measured at the indicated positive delay times. Negative-going signals correspond to parent ion depletion, while positive-going peaks indicate fragment ion formation.

Major fragment ions showing positive-going signals appear at *m*/*z* 91 for PB^+^, and at *m*/*z* 91, 92, and 57 for DMPB^+^. These are the same major fragment ions observed in the background mass spectra shown in Figure 2, suggesting that the thresholds for these dissociation channels lie below the probe photon energy, consistent with the thermochemical data in Table 1. Note that in the PB^+^ TRMS spectra, the *m*/*z* 92 signal remains no greater than the expected ^13^C peak intensity for C_7_H_7_^+^ ion, as seen in the background mass spectra in Figure 2, indicating that the formation of C_7_H_8_^+^ remain negligible upon photodissociation of PB^+^ by the visible probe photon.^85^

In addition to the major fragment ions, the TRMS spectra also reveal several lower-mass fragment ions with lower intensities. As described in Section 3.1, the UV laser pulse energy dependence indicates that production of these lighter fragment ions requires absorption of more than three UV photons. Therefore, photodissociation of ground-state parent cations by a single visible probe photon is energetically insufficient to produce these lighter fragment ions. This consideration would suggest that their formation may involve two-photon absorption of the probe pulse. However, this speculation is not supported by the observations that these lighter fragment ions persist with similar relative intensities to the major ones in TRMS spectra recorded using probe pulse energies varied over an 8-fold range from 0.3 to 2.4 μJ/pulse (see Figure S4), indicating that their production is more likely caused by one-photon absorption of the probe. On the other hand, when both pump and probe pulse energies are proportionally reduced by a factor of 3, the signals of lighter fragment ions become negligible compared to the major ones, as shown in Figure 9.

**Figure 9:**
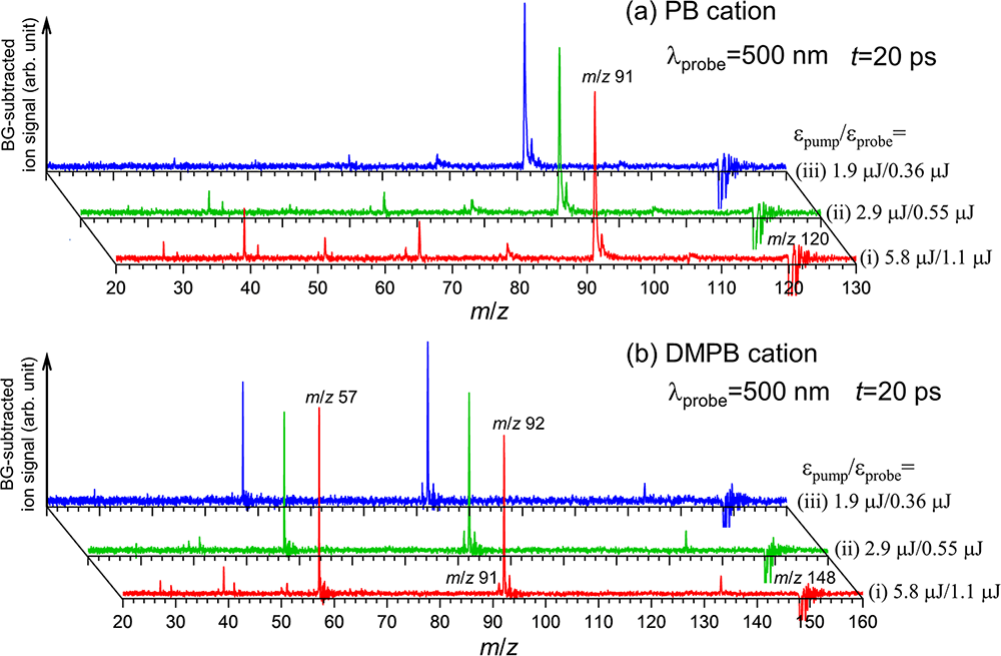
Background-subtracted time-resolved mass spectra of (a) PB^+^ and (b) DMPB^+^ measured at λ_probe_ = 500 nm and a fixed delay time of Δ*t* = 20 ps, with both pump and probe pulse energies proportionally reduced by factors of about two and three from those used under typical conditions (trace (i)). For clarity, the parent ion depletion signal has been truncated, and each spectrum is normalized to its most intense fragment-ion peak. Note that when both pump and probe pulse energies are reduced by a factor of 3 (trace (iii)), the signals of lighter fragment ions become negligible compared to the major ones.

These observations suggest that the lighter fragment ions observed in the TRMS spectra originate from excited states of parent cations produced at the 3*h*ν_uv_ level via ladder climbing within the pump pulse, followed by one-photon absorption of the probe. As shown in Figure 2, photoionization of PB and DMPB with a UV pulse energy of ∼5.8 μJ produces a substantial amount of the major fragment ions, indicating that a significant fraction of parent ions is produced at the 3*h*ν_uv_ level. These cationic excited states possess sufficient energies to yield the major fragment ions but not the lighter ones. However, additional absorption of a probe photon prior to the decay of these cationic excited states makes dissociation pathways leading to lighter fragment ions energetically accessible. These dynamics are further examined through analysis of fragment ion transients, as described below.

### 3.6 Fragment Ion Transients

Since fragment ions are formed via photodissociation of parent ions induced by the probe pulse, their transients are expected to exhibit temporal behavior complementary to that of the parent ion depletion transients, that is, similar temporal evolution but with opposite sign, as demonstrated in previous studies.^18,20^ This complementary relationship is indeed observed for PB^+^. As shown in Figure 10a, the major fragment ion (*m*/*z* 91, C_7_H_7_^+^) transient, measured at λ_probe_ = 500 nm under the same condition, displays a positive-going, time-independent signal after time zero, mirroring the PB parent ion depletion transient shown in Figure 6.

**Figure 10:**
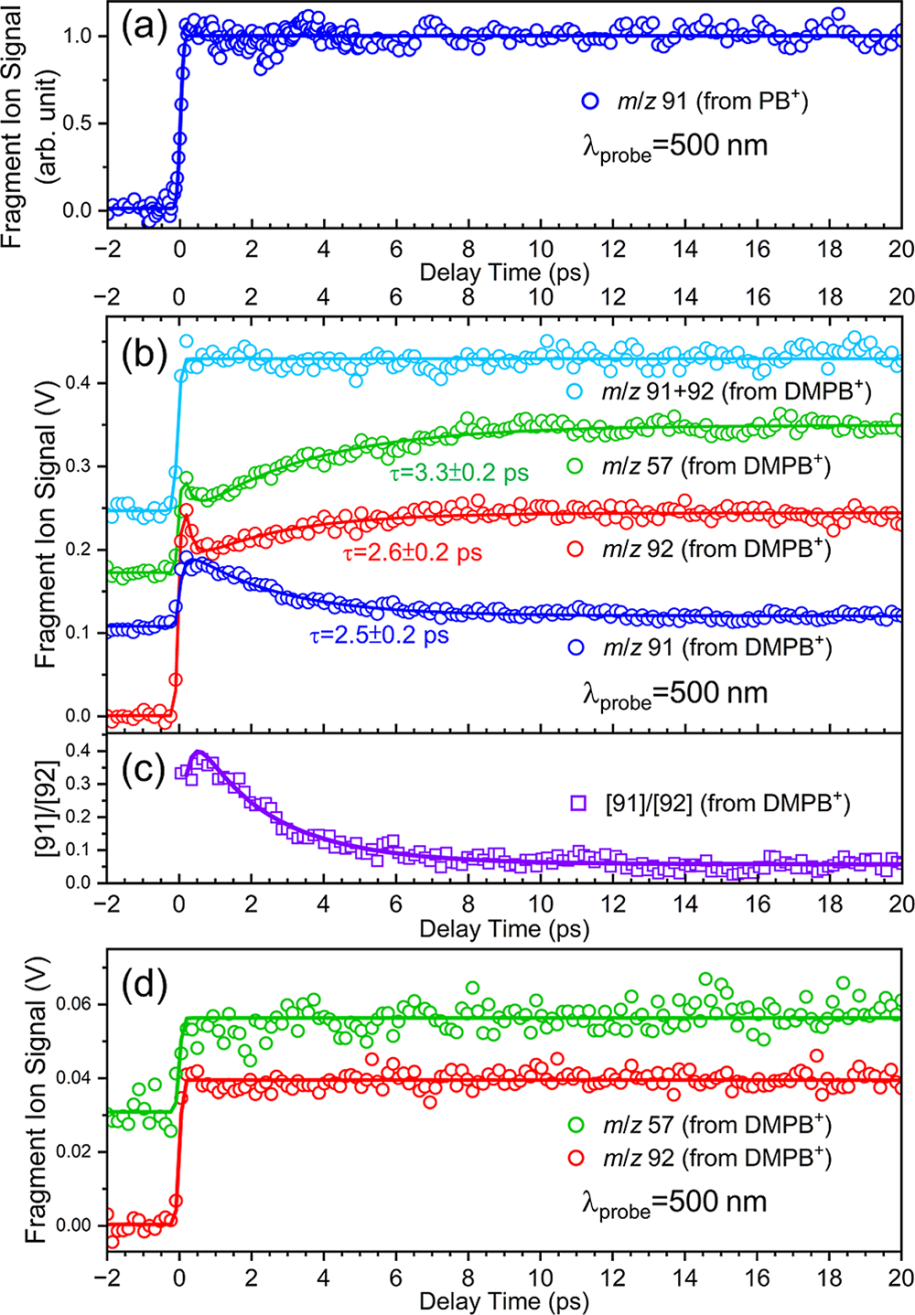
(a) Fragment ion formation transient of *m*/*z* 91 from PB^+^. (b) Fragment ion formation transients of major fragment ions from DMPB^+^: *m*/*z* 57 (green open circles), 91 (blue open circles), and 92 (red open circles). The transient labeled *m*/*z* 91 + 92 (light blue open circles) was obtained by gating *m*/*z* 91 and 92 ion signals together. The pump and probe pulse energies in (a,b) are the same as those used in Figure 6. (c) Time-dependent ratio of the integrated *m*/*z* 91 to *m*/*z* 92 fragment ion signals, derived from data shown in (b) with background subtracted. (d) Fragment ion formation transients of *m*/*z* 57 and 92 from DMPB^+^, with both pump and probe pulse energies reduced by a factor of 3 relative to those used in (b). All transients in this figure are measured at λ_probe_ = 500 nm. Solid lines are the best fits to the data (open circles) to either multiexponential or step-function models.

However, for DMPB^+^, the major fragment ions at *m*/*z* 92, 91, and 57 exhibit temporal behaviors that differ markedly from those of the parent ion depletion transients, as shown in Figure 10b. The *m*/*z* 92 and 57 fragment ion transients show similar rise components of ∼2–3 ps, whereas the *m*/*z* 91 transient displays a decay component with a time constant and amplitude nearly identical to the rise observed in the *m*/*z* 92 transient. Notably, as also shown in Figure 10b, the transient recorded by gating both *m*/*z* 91 and 92 ion signals together exhibits no discernible temporal variation beyond time zero. These observations suggest that the underlying dynamics leading to the formation of *m*/*z* 91 and 92 ions are competitive, resulting in their complementary transient signals. Transients of lighter fragment ions are more difficult to measure due to lower S/N ratios; however, they all appear to exhibit temporal behaviors similar to those of the *m*/*z* 91 or 92 fragments, as inferred from TRMS spectra shown in Figure 8. The interpretation of these observations is discussed in Section 4.5.

### 3.7 Time-Resolved Ion Photofragmentation Spectra

The parent ion depletion yield is expected to be governed by both the resonance absorption cross section and photofragmentation yield of the parent ion at a specific probe wavelength. To investigate its probe wavelength dependence, Φ­(λ), we measured parent ion depletion yields using the procedure described in Section 3.2 at various probe wavelengths in the visible spectral region, at a fixed delay time of 10 ps. The number of probe photons per pulse was kept constant at ∼1.45 × 10^12^ photons/pulse across the 400–800 nm visible spectral range by adjusting the pulse energy from 0.72 to 0.36 μJ/pulse accordingly. For each wavelength, ion depletion yield measurements were repeated multiple times, and on different days in some cases, to confirm reproducibility.

The results, shown in Figure 11a,b for PB^+^ and DMPB^+^, respectively, represent ultrafast time-resolved photofragmentation (TRPF) spectra measured with a temporal resolution of ∼100 fs at a fixed delay time of 10 ps. Note that the blue lines connecting the data points are included solely as visual guides to the overall spectral profile; finer spectral features may not be resolved due to the relatively large spectral sampling interval (∼25 nm) and the probe pulse bandwidth (∼2–8 nm across the 400–800 nm spectral region).

**Figure 11:**
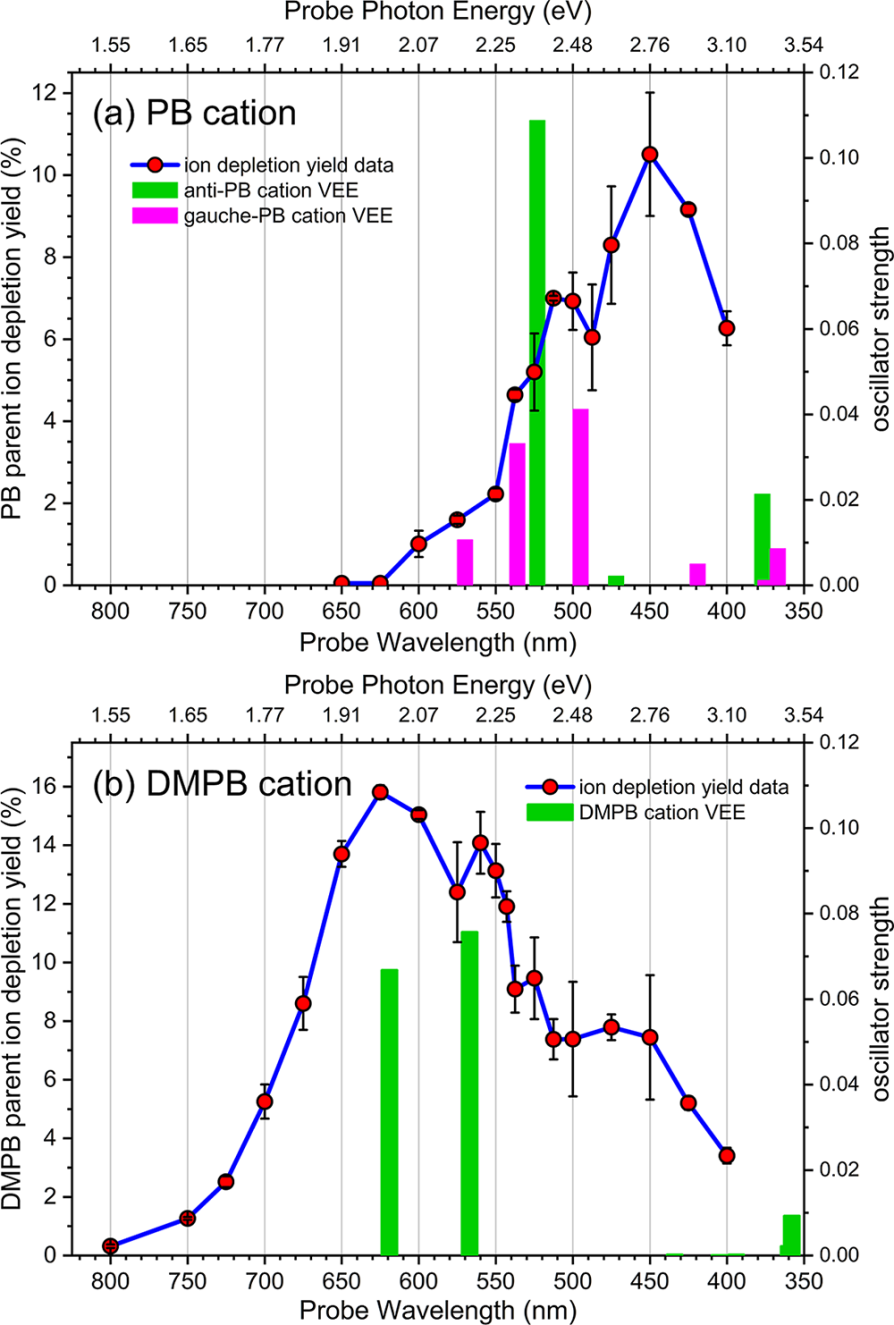
Ultrafast time-resolved photofragmentation (TRPF) spectra measured at a fixed pump–probe delay time of 10 ps for (a) PB and (b) DMPB cations. Parent ion depletion yields were measured at various probe wavelengths with the number of probe photons per pulse (∼1.45 × 10^12^ photon/pulse) kept constant by adjusting the probe pulse energy accordingly. Each data point (red solid circles) represents the average of multiple measurements using the procedure described in Section 3.2, and the error bars denote one standard deviation. The blue connecting lines are included as visual guides to the spectral profiles. Solid bars represent vertical excitation transitions with calculated VEEs below 3.5 eV at the optimized geometries of cationic ground states, as described in Section 3.8. The positions of the bars correspond to vertical excitation wavelengths (lower axis) and energies (upper axis), and their lengths are proportional to the calculated oscillator strengths (right axis). In panel (a), green and pink solid bars represent transitions of the *anti*- and *gauche*-PB^+^ conformers, respectively.

In general, a two-dimensional TRPF spectrum, Φ­(λ, *t*), can be constructed by combining the time-resolved spectral profile of Φ­(λ, 10 ps), shown in Figure 11, with parent ion depletion transients recorded at various probe wavelengths. However, as shown in Section 3.4, for both PB^+^ and DMPB^+^, the ion depletion transients measured at various probe wavelengths spanning their respective TRPF spectra exhibit no observable temporal evolution following the initial ultrafast rise (<100 fs). Consequently, the TRPF spectra remain essentially time invariant, and the spectra shown in Figure 11a,b are representative of TRPF spectra at all later delay times. Nevertheless, it should be pointed out that in other ionic systems undergoing significant relaxation after photoionization, TRPF spectra may exhibit pronounced time dependence, which can provide valuable insights into the transient species involved in the relaxation process.^20^

The TRPF spectrum of PB^+^ exhibits a maximum at ∼450 nm, whereas that of the DMPB cation peaks at a significantly longer wavelength, around 650 nm, and appears broader in shape. These TRPF spectra are presumably determined by the product of the wavelength dependence of ion photofragmentation yield and that of the ionic resonance absorption cross section. According to the thermochemical data presented in Table 1, the lowest dissociation limits for PB^+^ and DMPB^+^ are 1.56 eV (795 nm) and 1.38 eV (898 nm), respectively. Above these dissociation limits,^94^ the ion fragmentation yield is expected to rapidly approach unity. Therefore, within the spectral range studied, the TRPF spectra primarily reflect the resonance absorption spectra of the parent cations in their ground state.

Alkylbenzene cations have been extensively studied using ion cyclotron resonance photodissociation spectroscopy, which revealed prominent resonance absorptions in the UV–visible spectral region.^95,96^ The photodissociation spectrum of PB^+^ was found to peak at 2.65 eV (468 nm),^96^ in excellent agreement with the present results. A redshift in the absorption spectrum with increasing alkyl chain length has also been observed,^95^ consistent with the spectral shift observed between PB^+^ and DMPB^+^ spectra shown in Figure 11.

No prior photodissociation spectrum has been reported for the DMPB cation. However, a previous He­(I) photoelectron spectroscopy (PES) study^77^ determined the four lowest IEs of DMPB to be 8.77, 9.13, 10.73, and 11.41 eV, corresponding to the cationic ground state (D_0_) and three low-lying excited states (D_1_, D_2_, and D_3_), respectively. The ∼ 2 eV (620 nm) energy gap between the D_0_ and D_2_ states is consistent with the observed 650 nm peak in the TRPF spectrum of the DMPB cation. TDDFT calculations for both cations, presented below, predict resonance absorption transitions in spectral regions consistent with the TRFP spectra observed in this study.

### 3.8 Quantum Chemistry Computations

To investigate the energetics relevant to photoionization and subsequent relaxation in PB and DMPB, quantum chemistry calculations were performed using the Gaussian 16 program package.^97^ Optimized geometries of the neutral and cationic ground states of both PB and DMPB, obtained from CBS-QB3 calculations, are presented in Figure 12. The corresponding Cartesian coordinates are given in the Supporting Information. C–C bond lengths are annotated in the figure adjacent to their respective bonds. Calculated relative energies and IEs are summarized in Table 2.

**Figure 12:**
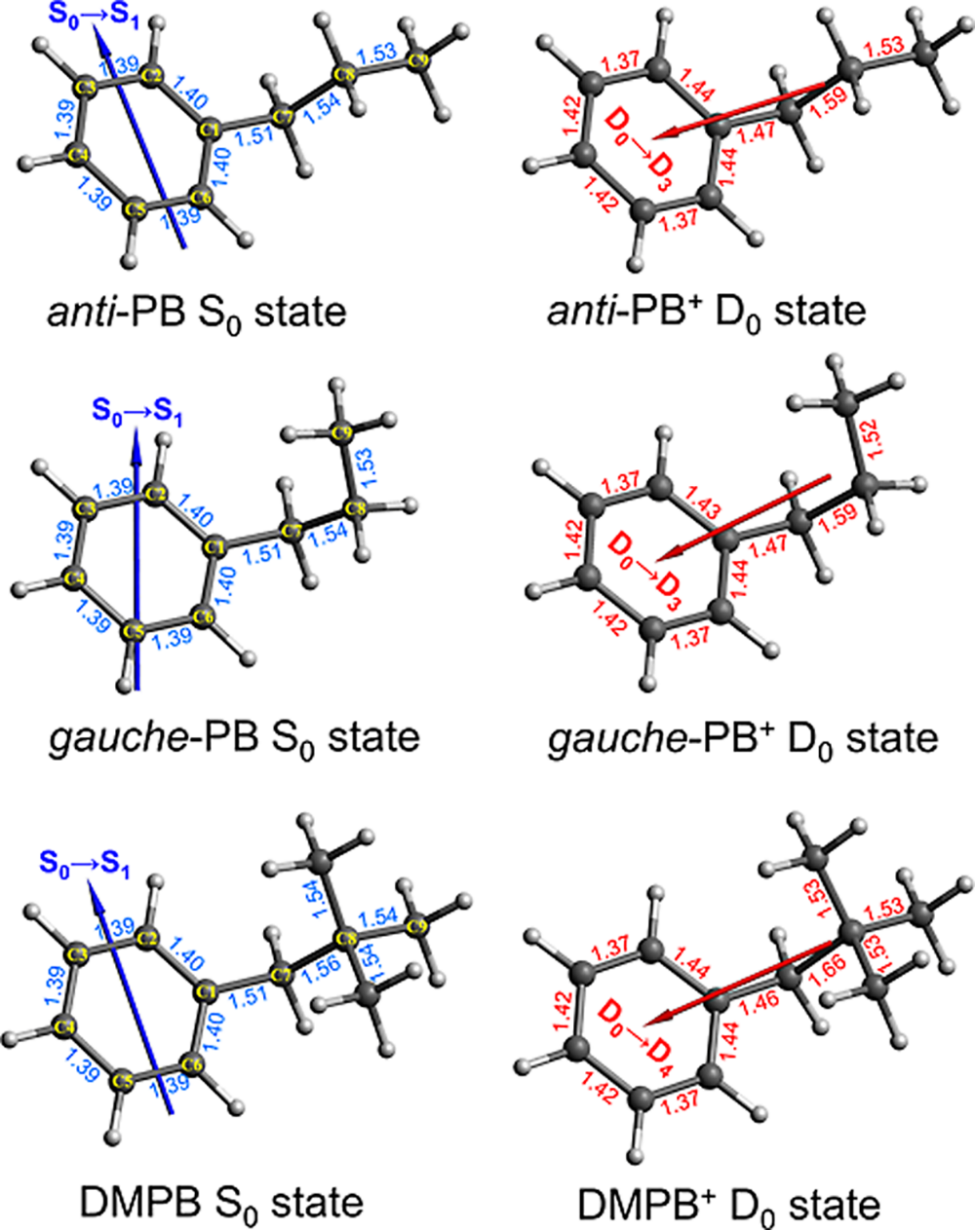
Optimized structures of the neutral and cationic ground states of PB and DMPB, obtained from CBS-QB3 calculations. C–C bond lengths (in Å) are indicated near the corresponding bonds. Blue arrows overlaid on the neutral structures represent the transition dipole moment vector (not to scale) for the S_0_ → S_1_ transition, while red arrows on the cationic structures denote the transition dipole moment vectors (not to scale) for the D_0_ → D_3_ transition in PB^+^ and the D_0_ → D_4_ transition in DMPB^+^.

**Table 2:** Relative Energies and Ionization Energies of PB Conformers and DMPB Calculated at the CBS-QB3 Level of Theory

	CBS-QB3				exp. values
	Δ*E*(*S*_0_)^a^/cm^–1^	Δ*E*(D_0_)^b^/cm^–1^	AIE^c^ (eV)	VIE^d^ (eV)	IE (eV)
*anti*-PB	0	0	8.794	9.024	8.71^e^
*gauche*-PB	37	127	8.805	9.036	8.73^e^
DMPB			8.728	8.963	8.77^f^

a Zero-point energy (ZPE) corrected relative energies calculated with CBS-QB3 between *anti*- and *gauche*-PB conformers in their neutral ground states (S_0_).

b ZPE corrected relative energies calculated with CBS-QB3 between *anti*- and *gauche*-PB^+^ conformers in their cationic ground states (D_0_).

c Adiabatic ionization energies calculated with CBS-QB3.

d Vertical ionization energies calculated with CBS-QB3, as described in the text.

e From refs 74 and 75 .

f From ref 77.

For neutral DMPB, only one stable conformer was found, in agreement with an earlier spectroscopic investigation.^76^ In contrast, two stable conformers were identified for neutral PB, as shown in Figure 12. The *anit*-PB conformer adopts a fully extended alkyl chain oriented away from the phenyl ring, whereas in the *gauche*-PB conformer the chain folds back toward the ring. At the CBS-QB3 level of theory, the *anti* conformer is predicted to be slightly more stable than the *gauche* form by only ∼37 cm^–1^ in the neutral ground state (S_0_), suggesting that both conformers are likely to be comparably populated in the molecular beam,^98^ consistent with previous spectroscopic observations.^74,75^ In the cationic ground state (D_0_), the *anti*-PB^+^ conformer becomes even more stable than the *gauche*-PB^+^ conformer by about 127 cm^–1^. These results indicate that ionization induces only a minor change in the relative stability between the *anti*- and *gauche*-PB conformers.

As summarized in Table 2, the calculated adiabatic IEs are ∼8.8 eV for both PB conformers and ∼8.73 eV for DMPB, in good agreement (<0.1 eV) with the experimental values. Vertical IEs were determined as the difference between the CBS-QB3 single-point energies of the cationic and neutral ground states, calculated at the neutral optimized geometries. The results indicate that the energy differences between the vertical and adiabatic IEs are about 0.23 eV for both PB and DMPB. Accordingly, femtosecond 1 + 1 REMPI with a total photon energy of ∼9.3 eV is expected to produce an ensemble of ground-state cations with a vibrational energy distribution peaking around 0.23 eV (∼1855 cm^–1^). Previous two-color 1 + 1′ REMPI PES studies of PB and ethylbenzene indeed showed pronounced vibrational structures extending to excess energies of at least 0.2 eV.^75,99^ This vibrational energy is expected to be initially deposited in several Franck–Condon (FC) active modes associated with significant structural changes upon ionization, most notably in the C–C bond lengths within the phenyl ring and along the C1–C7–C8 carbon chain, as shown in Figure 12. All torsional angles in the alkyl groups remain nearly unchanged, resulting in minimal conformational changes upon ionization.

To evaluate the UV resonance absorption of nascent cations within the pump pulse, vertical excitation energies (VEEs) of cations were calculated at the optimized structures of the neutral ground states at the TD-B3LYP/cc-pVTZ level of theory. The results, referenced to the neutral ground-state minimum and summarized in Table S1, correspond to energies of excited states at the initial D_0_-state geometries, prior to any structural relaxation following vertical ionization. A schematic illustration of the cationic excited-state manifold, along with the relevant neutral states involved in the 1 + 1 REMPI process, is shown in Figure 13. Both PB and DMPB cations exhibit a high density of electronic states, with several excited states possessing appreciable oscillator strengths near the UV pump photon energy employed in this study. These ionic resonances are expected to greatly enhance the ladder-climbing mechanism following ionization.^44–48^ Consequently, absorption of an additional UV photon by the nascent cation produced via femtosecond 1 + 1 REMPI within the pump pulse, promoting it to higher excited states at the 3*h*ν_uv_ level, is expected to occur readily even under moderate irradiances. This interpretation is supported by the laser irradiance dependence of mass spectra presented in Section 3.1.

**Figure 13:**
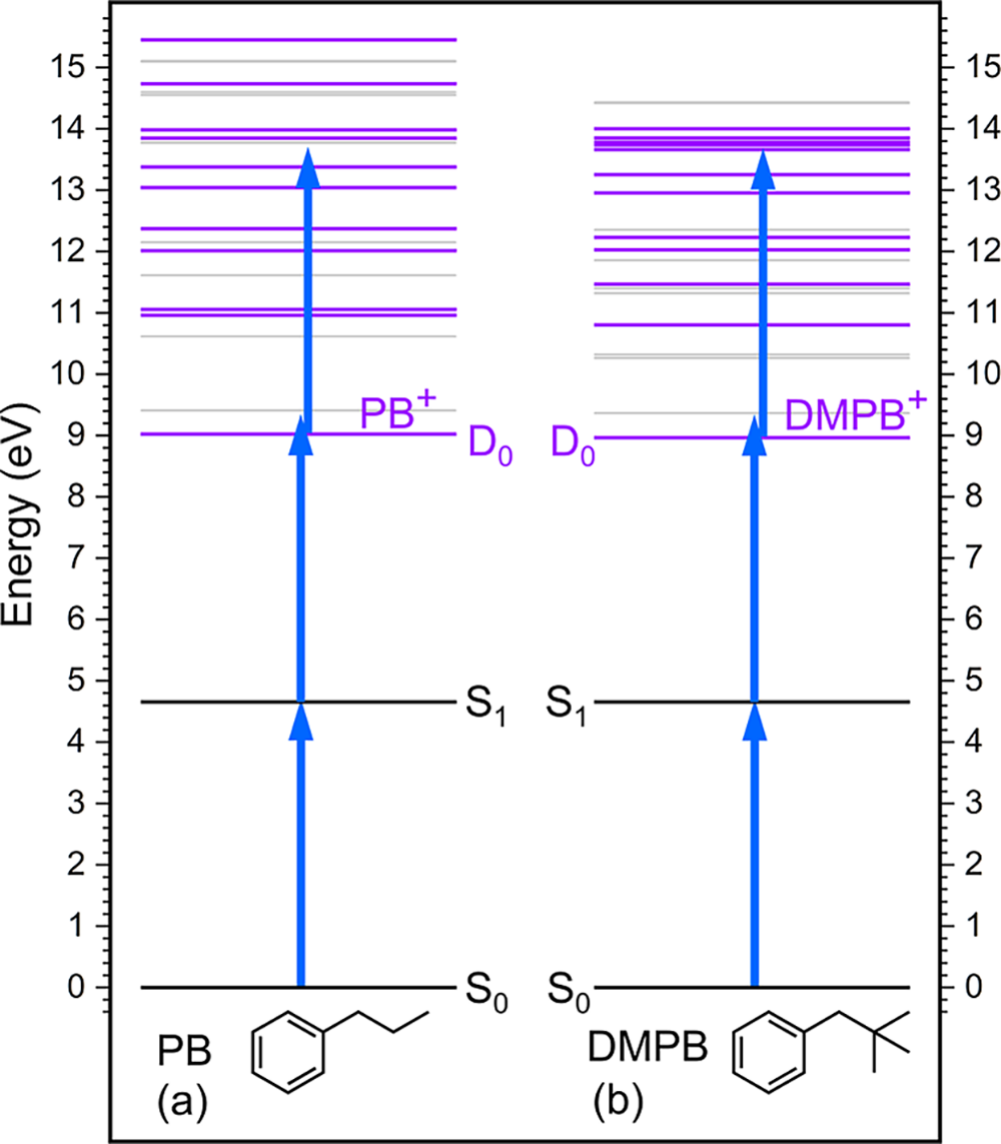
Schematic representation of the cationic excited-state manifolds of (a) *anti*-PB and (b) DMPB, calculated at the TD-B3LYP/cc-pVTZ level at the optimized structure of the neutral ground state. Horizontal gray lines denote the cationic electronic states, with energies referenced to the neutral S_0_-state minimum (see data in Table S1). States exhibiting oscillator strengths greater than 0.001 for transitions from the D_0_ state are colored in purple to highlight efficient postionization resonance absorptions. The neutral S_0_ and S_1_ states involved in the 1 + 1 REMPI process are shown as black lines. Vertical blue arrows, drawn to scale in length, represent the UV pump photon energy. The third UV photon indicates the postionization resonance absorption by the nascent cation within the pump pulse.

VEEs of cations were also calculated at the optimized structures of the cationic ground states at the same level of theory. The results, summarized in Table 3 for states with excitation energies below 4 eV, represent the resonance absorption energies of the relaxed ground-state cation in the visible spectral region probed in this study. These results will be discussed further in the following section.

**Table 3:** Vertical Excitation Energies, Wavelengths, and Oscillator Strengths Calculated at the TD-B3LYP/Cc-pVTZ Level of Theory at Optimized Structures of PB^+^ and DMPB^+^ Ground States

	*anti*-PB^+^			*gauche*-PB^+^			DMPB^+^		
	VEE at D_0_ min			VEE at D_0_ min			VEE at D_0_ min		
states	Δ*E* ^a^ (eV)	λ^b^ (nm)	osc^c^	Δ*E* ^a^ (eV)	λ^b^ (nm)	osc^c^	Δ*E* ^a^ (eV)	λ^b^ (nm)	osc^c^
D_0_	0			0			0		
D_1_	0.908	1365	0.0001	0.888	1396	0.0001	0.941	1318	0.0001
D_2_	2.246	552	0.0000	2.177	570	0.0107	2.004	619	0.0668
D_3_	2.369	523	0.1088	2.315	536	0.0332	2.172	571	0.0000
D_4_	2.627	472	0.0022	2.506	495	0.0412	2.188	567	0.0757
D_5_	2.889	429	0.0000	2.963	419	0.0050	2.858	434	0.0002
D_6_	3.289	377	0.0214	3.308	375	0.0012	3.064	405	0.0001
D_7_	3.685	336	0.0001	3.377	367	0.0086	3.151	394	0.0002
D_8_	3.709	334	0.0183	3.970	312	0.0009	3.444	360	0.0022
D_9_							3.466	358	0.0093
D_10_							3.671	338	0.0102

a Vertical excitation energy (VEE) of cations calculated at the optimized structure of the D0 state.

b Corresponding wavelength of VEE.

c Calculated oscillator strength for the corresponding D0 → Dn transition.

## 4 Discussion

### 4.1 Initial States Responsible for the Parent Ion Depletion Transient Signals

We begin by considering the initial states responsible for the observed parent ion depletion transient signals. As noted in Sections 3.1 and 3.8, at the typical UV pump pulse energy of ∼5.8 μJ/pulse used in this work, absorption of a third UV photon by the nascent cation within the pump pulse is efficient, owing to the high density of electronic states in alkylbenzene cations in the UV spectral region. Consequently, the initial parent cation ensemble is primarily composed of a mixture of cations produced at the 2*h*ν_uv_ and 3*h*ν_uv_ levels.

As schematically illustrated in Figure 14, absorption of a third UV photon by the nascent cation within the pump pulse promotes the system to high-lying excited states (D_n*_) with internal energies (≥4.65 eV) well above the dissociation limits for the major fragment ions (see Table 1). These high-lying D_n*_ states are therefore expected to undergo either adiabatic or nonadiabatic transitions leading to dissociation. The only pathway by which a cation can survive dissociation after excitation to D_n*_ states is via radiative transitions to lower electronic states below the dissociation limits. However, as shown in Figure 13 and Tables S1 and S2, there are numerous states of the same multiplicity below the D_n*_ states, and the small energy gaps among these states warrant rapid sequential internal conversions that ultimately lead to the ground state, where slow unimolecular dissociations can occur. As a result, the radiative quantum yields of the D_n*_ states are likely negligible, making the high-lying D_n*_ states at the 3*h*ν_uv_ level dissociate with near-unity yields via either adiabatic or nonadiabatic pathways. Therefore, although these high-lying excited states may exhibit a range of ultrafast lifetimes, their dynamics do not manifest in the parent ion depletion transient signals. This is because they dissociate almost completely, with or without additional probe photon energy, rendering subsequent probe absorption inconsequential to the parent ion signal. Such an advantage may not be realized when near-IR pump pulses are used under conditions where both MPI and tunnel ionization are operative, particularly if low-lying states below the dissociation limits are accessible via one-photon absorption in the near-IR.

**Figure 14:**
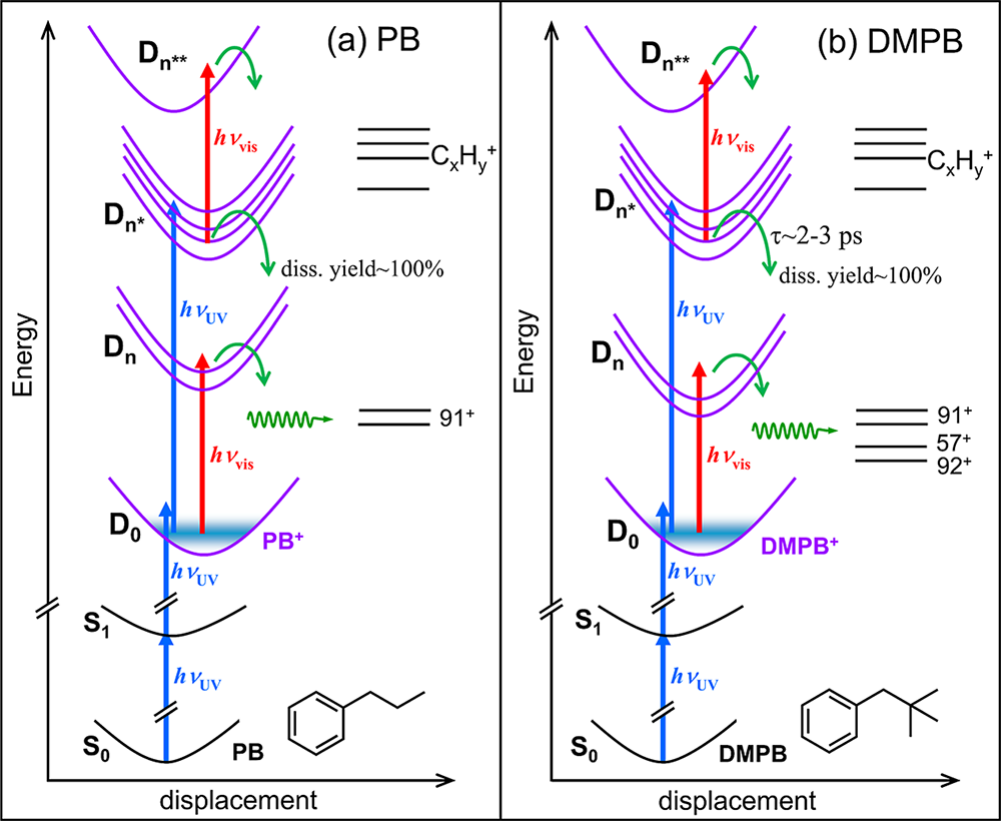
Schematic illustration of cationic states involved in the initial preparation of parent cations at the 2*h*ν_uv_ and 3*h*ν_uv_ levels within the UV pump pulse for (a) PB and (b) DMPB. Also depicted are the relevant ion fragmentation energetics and probe-induced transitions via ionic resonance absorption in the visible region that lead to fragmentation. Blue and red vertical arrows (not drawn to scale) represent the UV pump and visible probe photon energies, respectively. D_*n*_ denotes excited states that are resonant with the D_0_ ground state in the visible region and have total energies at the 2*h*ν_uv_ + *h*ν_vis_ level; D_n*_ represents states accessed at the 3*h*ν_uv_ level within the pump pulse; and D_n**_ indicates states at the 3*h*ν_uv_ + *h*ν_vis_ energy level. Green curved and wavy arrows denote nonadiabatic and/or adiabatic pathways leading to fragmentation. Asymptotic energy levels labeled 57^+^, 91^+^, and 92^+^ indicate the dissociation limits for the production of the major fragment ions (*m*/*z* 57, 91, and 92) and their respective neutral fragments. Levels labeled C_*x*_H_*y*_^+^, located at higher energies, correspond to dissociation limits for lighter fragment ions.

Accordingly, although the UV pump pulse used here produces an ensemble of cations with a wide range of total energies, we conclude that only parent ions produced at the 2*h*ν_uv_ level contribute to the observed parent ion depletion transient signals. On the other hand, while the high-lying cationic excited states produced at the 3*h*ν_uv_ level do not contribute to the parent ion depletion transients, they may influence the fragment ion transients, as discussed below.

### 4.2 Initial States Prepared at the 2*h*ν_uv_ Level

Nascent parent ions produced at the 2*h*ν_uv_ level may include those produced by 1 + 1 REMPI and delayed autoionization via neutral superexcited (SE) states lying above the first IE. We first consider cationic states accessed via direct ionization of the neutral S_1_ state in the 1 + 1 REMPI process. An early He­(I) PES study^77^ determined the four lowest IEs of DMPB to be 8.77, 9.13, 10.73, and 11.41 eV, corresponding to the cation ground state (D_0_) and the first three low-lying excited states (D_1_–D_3_), respectively. For PB, the first adiabatic IE has been reported to be about 8.7 eV for both conformers. Although experimental data on the excited states of the PB cation are not available, its second IE can be estimated based on those of ethylbenzene and *n*-butylbenzene, which have been reported as 9.29 and 9.24 eV, respectively.^100^ Thus, the second IE of PB, which correspond to its cationic D_1_ state, is likely to lie within this range. Hence, 1 + 1 REMPI with a total energy of ∼9.3 eV is energetically sufficient to populate not only the cationic ground state (D_0_) but also the first excited state (D_1_) for both PB^+^ and DMPB^+^.

In both molecules, the electron configuration of the neutral ground state is ...(π_H‑1_)^2^(π_H_)^2^, where π_H_ and π_H‑1_ represent the highest and second-highest occupied molecular orbitals, respectively. In the 1 + 1 REMPI process of alkylbenzenes, ionization proceeds via the neutral S_1_ state, which can be described as ...(π_H‑1_)^2^(π_H_)^1^(π_L_)^1^, where π_L_ denotes the lowest unoccupied molecular orbital. Ionization from the S_1_ state to the cation D_0_ state occurs readily via one-electron ejection from the π_L_ orbital. In contrast, formation of cations in the D_1_ state, which has a configuration of ...(π_H‑1_)^1^(π_H_)^2^, requires ejection of an electron from the π_H‑1_ orbital accompanied by an electron transition from π_L_ to π_H_. Such one-photon, two-electron processes are formally forbidden and typically inefficient, though they may become weakly allowed through configuration interaction in some cases.^101^

Therefore, unlike single-photon ionization or nonresonant MPI, 1 + 1 REMPI of alkylbenzenes via their S_1_ states exhibits a strong propensity for populating the cation D_0_ state, despite the fact that both D_0_ and D_1_ states are energetically accessible. Previous 1 + 1 REMPI PES studies of toluene and ethylbenzene^99^ via the S_1_ state reported no evidence of D_1_ state production, further supporting the preference for D_0_ state formation. As such, production of the D_1_ state is likely minimal in the present case. However, if formed, the D_1_ state may undergo rapid internal conversion to the D_0_ state and give rise to a minor temporal component in the parent ion depletion transients, provided that the D_1_ and D_0_ states exhibit different photodissociation yields upon visible probe absorption. A recent SFI-PF study of toluene,^41^ employing 50 fs laser pulses at 800 nm as the ionization source, showed that the cationic D_1_ state can be populated via SFI and that D_1_ → D_0_ internal conversion occurs in 530 fs.^41^ In the present study, no temporal component on a comparable time scale was observed, suggesting that D_1_-state population via 1 + 1 REMPI is likely negligible for both PB^+^ and DMPB^+^.

We next consider SE states accessed via two-photon excitation within the UV pump pulse. SE states are neutral excited states that lie above the first IE of the molecule. They include highly excited valence states as well as Rydberg states with internally excited ion cores, both of which can couple to the isoenergetic ionization continuum and undergo rapid autoionization.

In the present study, Rydberg SE states that are resonant with the two-photon energy (∼9.3 eV) correspond to those with ion cores that are highly vibrationally excited, approximately 0.6 eV above the zero-point level. Consequently, these Rydberg SE states are expected to exhibit very low excitation probabilities due to poor FC overlaps with vibronic levels near the neutral S_1_-state origin. Even at exact resonance with a Rydberg SE state, prompt ion production typically dominates, as commonly observed in threshold ionization photoelectron and photoion studies.^102^ Thus, the population of Rydberg SE states is likely minimal in this study. Furthermore, even if an appreciable population were present, we argue that Rydberg-state autoionization dynamics would not contribute to the observed parent ion depletion transients, as the resonance absorption spectra of Rydberg-state ion cores and the resulting cations produced via autoionization are expected to be nearly identical. This same principle is employed in photoinduced Rydberg ionization (PIRI) spectroscopy, developed by Johnson’s group, for measuring electronic absorption spectra of molecular ions.^103^ Based on these considerations, we conclude that Rydberg SE states are not relevant to the present experiments and will not be discussed further.

Finally, we consider valence SE states, which typically arise from excitations from inner valence orbitals or two-electron promotions into higher-lying orbitals. These states are often observed in vacuum UV absorption spectra or photoionization efficiency (PIE) curves as sharp features superimposed on the continuum above the lowest ionization threshold. In the present experiments, excitation of such a state would require an accidental resonance with a valence SE state located at the two-photon energy (∼9.3 eV). If present, these states can be populated and may undergo rapid relaxation via coupling to the ionization continuum and/or to high-lying Rydberg states, ultimately leading to delayed autoionization and the production of ground-state cations.^104^ Such dynamics may manifest in the parent ion depletion transients, provided that the resonance absorption of the valence SE state and the cation ground state are different. In the present study, we exclude the involvement of valence SE states on the basis of the following evidence. First, a previous PIE study of hydrocarbons reported no discernible SE-state features in the PIE curve for PB within the 8.5–11.7 eV energy range.^105^ Second, a two-color 1 + 1′ REMPI PES study of ethylbenzene via the S_1_-state origin, at a total energy ∼1600 cm^–1^ above the ionization threshold, showed no evidence of autoionizing states.^99^

To summarize, the initial state responsible for the observed parent ion depletion transient signal in the present study is primarily the cationic ground state (D_0_) produced via direct ionization of the neutral S_1_ state in the 1 + 1 REMPI process. Although population of the D_1_ state is energetically accessible, its contribution is expected to be minimal due to the formally forbidden nature of the one-photon, two-electron transition required for its formation. Contributions from neutral valence and Rydberg SE states can be excluded based on the preceding discussion.

### 4.3 Cationic TRPF Spectra and Resonant Probe Transitions

As noted in Section 3.7, the TRPF spectra shown in Figure 11 remain time-invariant following the initial ultrafast relaxation (<100 fs) and closely resemble the resonance absorption spectra of cation ground states near equilibrium structures. Therefore, VEEs calculated at the optimized D_0_-state structures are expected to provide a better prediction of the TRPF spectra. The results of such calculations for PB^+^ and DMPB^+^, summarized in Table 3, indeed predict several electronic transitions with appreciable oscillator strengths in the visible region. The excited states corresponding to these transitions are denoted as the D_*n*_ states in Figure 14. For ease of comparison, transitions with calculated VEEs below 3.5 eV are represented in Figure 11 as solid bars, with lengths proportional to their oscillator strengths, overlaid on the experimental TRPF spectra.

For the DMPB cation, the calculated VEEs of two low-lying bright states closely match the observed maxima in the TRPF spectrum. In contrast, the predicted low-lying bright states for the PB cation deviate somewhat from the observed maximum of TRPF spectrum. Nonetheless, the bright states of DMPB^+^ are predicted to lie at lower energies than those of PB^+^, consistent with the experimentally observed redshift of the DMPB^+^ spectrum relative to that of PB^+^. In both cases, the observed spectral profiles likely reflect vibronic structures combined with the broad vibrational energy distributions in the cationic ground states produced by photoionization.

Time-dependent DFT (TDDFT) results presented in Table 3 indicate that resonance absorptions of the PB cation in the visible region originate primarily from the D_0_ → D_3_ transition of the *anti* conformer and from the D_0_ → D_3_ and D_0_ → D_4_ transitions of the *gauche* conformer. For the DMPB cation, visible-region absorptions are predicted to arise mainly from the D_0_ → D_2_ and D_0_ → D_4_ transitions. Examinations of the dominant molecular orbital excitations involved in these transitions (see Figure S5) reveal that the corresponding excited states exhibit significant charge-transfer (CT) character. This feature was first noted by Dunbar in one of his early studies,^95^ where comparisons between the photoelectron spectra of alkylbenzenes and their corresponding alkanes led to the conclusion that visible-region absorptions in alkylbenzene cations arise from CT transitions. Upon excitation of these CT states, partial electron density shifts from the alkyl groups toward the phenyl ring, resulting in a redistribution of ring-localized positive charge in the ground state toward the alkyl groups in the excited states.^95^

As discussed in Section 3.3, the polarization dependence observed in the parent ion depletion transients is consistent with an orthogonal orientation between the pump and probe transition dipole moments. The CT nature of the probe transitions, as described above, suggests that the corresponding transition dipole moments are oriented approximately along the CT direction, pointing from the alkyl group toward the phenyl ring. TDDFT-calculated transition dipole moment vectors for the major cationic visible probe transitions and the neutral S_0_ → S_1_ excitations are illustrated in Figure 12, overlaid on the molecular structures. The vector coordinates for the major visible transitions are given in the Supporting Information. Except in the case of *gauche*-PB^+^, the pump and probe transition dipole moments are indeed predicted to be orthogonal, consistent with the observed polarization dependence shown in Figure 5. For *gauche*-PB^+^, the angle between the pump and probe transition dipole vectors is about 110°, which is still expected to behave more like orthogonal transitions.

The time invariance of the TRPF spectra observed here for alkylbenzene cations is a notable feature that deserves further discussion. This behavior indicates that, following photoionization, the D_0_ states of alkylbenzene cations do not undergo relaxation processes that substantially alter their resonance absorption profile in the visible region. In alkylbenzene cations, the primary relaxation processes available after photoionization are intramolecular vibrational energy redistribution (IVR) and conformational relaxation. The former will be discussed in the next section, while the latter is addressed below.

In the case of DMPB, only one stable conformer exists in both the neutral and cationic ground states; therefore, no conformational relaxation is expected following photoionization. In contrast, as described in Section 3.8, the *anti*-PB conformer is predicted to be slightly more stable than *gauche*-PB by only about 37 cm^–1^ in the neutral ground state. Both conformers are comparably populated in the molecular beam and are equally excited, since their S_1_-state origins lie within the bandwidth of the pump laser. Upon ionization, this energetic preference of the *anti* conformer increases slightly to about 127 cm^–1^ in the cationic D_0_ state. The energy barriers separating the two PB^+^ conformers are expected to be comparable to those associated with typical alkyl chain rotations, on the order of 0.1–0.2 eV. CBS-QB3 calculations predicted that the average vibrational energy deposited in the D_0_ state is ∼0.23 eV, which is just enough to overcome these barriers. Consequently, following photoionization, a fraction of PB cations are expected to possess sufficient internal energy to explore the full conformational space and re-equilibrate between the two conformers, resulting in a very slow conformational relaxation that shifts the equilibrium population slightly toward the anti-PB^+^ conformer.

However, given the small changes in relative energies between the two conformers upon ionization, the resulting shift in equilibrium population is expected to be minor. Furthermore, as shown in Figure 11a, the low-lying bright states of the two PB^+^ conformers exhibit comparable total oscillator strengths and lie in similar spectral regions, suggesting that their resonance absorption profile in the visible region are nearly identical. Together, these factors render conformational relaxation effectively undetectable by the present detection method, resulting in the observed time-invariant TRPF spectra of the PB cation.

A low-frequency (∼45 cm^–1^) mode, attributed to torsion of the alkyl chain relative to the phenyl ring, has been identified as FC active upon ionization in a previous 1 + 1′ REMPI PES study of the *gauche*-PB conformer.^75^ Although the pump pulse used in this study has a sufficient coherent bandwidth to excite a vibrational wave packet along this low-frequency torsional coordinate, no discernible oscillatory signals are observed in the parent ion depletion transients. This is probably not surprising, as the ring-to-chain torsional motion is expected to have minimal influence on the cation’s resonance absorption and is therefore not detectable by the present method. Other FC-active modes, such phenyl ring in-plane deformations and alkyl C–C stretches, have vibrational frequencies of at least several hundreds of cm^–1^. As such, the pump pulse used here does not have a sufficient bandwidth to coherently excite these modes.

### 4.4 Early-Time Behaviors of Parent Ion Depletion Transients

As shown in Figure 7, all parent ion depletion transients measured in this study exhibit an ultrafast initial rise on the order of several tens of femtoseconds. As discussed in Section 4.1, delayed ionization from SE states can be ruled out, and the initial population responsible for parent ion depletion signals is attributed predominantly to ground-state cations formed via direct photoionization of the S_1_ state in the 1 + 1 REMPI process. Recent advances in attosecond spectroscopy have revealed that photoionization time delays typically occur on time scales ranging from tens of attoseconds to a few femtoseconds.^106–111^ Similar results have been reported for REMPI processes;^110,111^ for example, a recent study on 1 + 2 REMPI of potassium atoms observed a resonant ionization delay of approximately 1 fs.^111^ Therefore, for practical purposes, the production of prompt ions via direct ionization of the S_1_ state in 1 + 1 REMPI can be regarded as instantaneous relative to the temporal resolution of this study.

Accordingly, the observed ultrafast rise in the parent ion depletion signal must reflect a rapid increase in resonance absorption strengths arising from subsequent relaxation following the instantaneous formation of cations. In the simple alkylbenzene cations studied here, such relaxation processes may include IVR and conformational relaxation. As discussed in Section 4.3, conformational relaxation involves overcoming energy barriers of ∼0.1–0.2 eV and is expected to occur on much longer time scales. These considerations led us to attribute the observed ultrafast initial rise to IVR occurring immediately after photoionization.

In molecules of comparable size and energy to the present systems, IVR has been shown to occur over several different time scales ranging from ∼100 fs to hundreds of picoseconds or longer.^90,112–116^ A well-established model for describing such behavior is the tier model of IVR dynamics,^114–116^ in which dark states are hierarchically organized into “tiers” according to their vibrational coupling strengths with the zero-order bright state. We propose that the ultrafast rise observed here arises from the initial vibrational dephasing that marks the onset of the IVR process.

As discussed in Section 3.8, upon photoionization a vibrational energy distribution peaking around 0.23 eV (∼1850 cm^–1^) is initially deposited in a limited number of FC-active modes in the D_0_ state. At the average vibrational energy of ∼ 1850 cm^–1^, the densities of vibrational states for PB^+^ and DMPB^+^ are estimated to be 7.8 × 10^2^ and 4.9 × 10^4^/cm^–1^, respectively.^117^ Consequently, photoionization by the UV pump pulse can prepare a coherent superposition of a large number of vibrational eigenstates within the ∼120 cm^–1^ coherent bandwidth. Each of these eigenstates arises from strong coupling between tier-one dark states with one of several zeroth-order FC-active states. The initially prepared nonstationary state then undergoes rapid vibrational dephasing, followed by slower, dissipative redistributions toward randomization, according to the tier model of IVR dynamics in large molecules.^115,116^ Therefore, the observed ≤100 fs initial rise is likely a manifestation of this early time dephasing governed by the pump bandwidth and vibrational coupling strengths.

As noted in Section 4.3, the probe transitions correspond to excitations from the cation D_0_ state to a few excited states (D_*n*_) with significant CT character, which reduce the partial positive charge on the phenyl ring. This suggests that the FC-active modes involved in photoionization are also likely to be active in the probe transitions. In spectral regions near the VEEs of these probe transitions, D_0_-state populations with high vibrational excitation in FC-active modes generally exhibit poor FC overlap with the D_*n*_ states, whereas those with low vibrational excitation typically display greater overlap. For cations with high vibrational excitation, a substantial portion of energy is initially localized in several FC-active modes. Rapid vibrational dephasing followed by dissipative IVR lowers the energy content in each FC-active mode, thereby enhancing absorption intensity near the VEEs. In contrast, for cations with low vibrational excitation, the limited initial energy in FC-active mode renders IVR inconsequential to the resonance absorption intensity.

In other words, cation populations with low vibrational excitation tend to exhibit an instantaneous rise in ion depletion transient, while those with high vibrational excitation require IVR to enhance resonance absorption. Such behavior is expected to be most pronounced in spectral regions near the VEEs, where the depletion yields are high.^118^ Based on these considerations, we propose that the initial temporal behavior of the parent ion depletion transients can be approximately described by a model function (eq 3) comprising an instantaneous rise, i.e., a step function, for ions produced with low vibrational excitation, and an exponential rise with a time constant τ_1_ for those produced with higher vibrational energies.

3 $$M\left(t\right)=A_{0}+A_{1}\left(1-e^{-t/\tau_{1}}\right),t\geq 0$$

Note that this model function is mathematically equivalent to a two-component reaction kinetic model. While dividing a continuous and broad vibrational energy distribution into only two ensembles is a simplified approximation, the model fits the experimental data well, as shown in Figures 7 and S1. The results indicate that the initial rise times for the DMPB cation (∼50–95 fs) are slightly shorter than those for the PB cation (∼60–110 fs), supporting our assignment that the observed dynamics arise from IVR, as the higher density of states in DMPB^+^ translates to a greater number of coherently excited vibrational eigenstates. However, the very limited increase in dephasing rate from PB^+^ to DMPB^+^ also implies weak coupling between FC-active modes and the low-frequency modes of the *tert*-butyl group.^119,120^

Although initial vibrational dephasing effectively accounts for the early time behavior, alternative explanations cannot be entirely ruled out. Specifically, a two-level system modeled using the optical Bloch equations in the coherent limit predicts that the half-maximum of the pump–probe transient signal is delayed by 0.327τ_p_ from time zero, where τ_p_ is the pump pulse duration (fwhm).^14,121^ It has also been shown that coherent excitation of a quasi-continuum system closely resembles incoherent excitation of a two-level system, wherein the transient signal reaches half-maximum at time zero.^14,121^ In the present case, the pump pulse first excites the S_1_ origin level, followed by ionization into the continuum, and therefore, this model would predict a delay of about 40 fs for the pump pulses used here (τ_p_∼120 fs). Thus, it is likely that resonant coherent excitation may partially contribute to the observed delay in the ion depletion transients. However, the fact that a longer rise time is consistently observed for PB^+^ relative to DMPB^+^ suggests that this effect alone cannot fully account for the initial ultrafast rise.

### 4.5 Fragment Ion Formation Transients

Since the parent ion depletion signal arises from photodissociation of parent cations induced by the probe pulse, fragment ion formation transients are expected to exhibit temporal behavior complementary to that of the parent ion depletion transients.^18,20^ This complementary relationship is observed for PB^+^ (see Figure 10a), but not for DMPB^+^. As shown in Figure 10b, fragment ion transients measured at *m*/*z* 92, 91, and 57 exhibit temporal components that are not present in the DMPB parent ion depletion transients. These observations suggest that, for DMPB^+^, the initial cationic ensemble underlying the fragment ion transients is not identical to that responsible for the parent ion depletion transients. Since parent ion depletion transients reflect dynamics of cations produced exclusively at the 2*h*ν_uv_ level and the UV pump pulse energy (∼5.8 μJ/pulse) used here produced an initial cationic ensemble at both the 2*h*ν_uv_ and 3*h*ν_uv_ levels (see Section 3.1), it is likely that the additional temporal components observed in the fragment ion transients arise from the cationic states formed at the 3*h*ν_uv_ level.

As illustrated in Figure 14b, although absorption of a probe photon by cationic states initially populated at the 3*h*ν_uv_ level (D_n*_) promotes the ion to even higher excited states (D_n**_), it does not alter the total parent ion dissociation yield, which is already nearly unity in the D_n*_ states. However, it may influence the fragmentation branching ratios among different dissociation pathways, owing to the energy dependence of fragment-ion branching ratios. As a result, the dynamics of D_n*_ states populated at the 3*h*ν_uv_ level can appear in the fragment ion transients, alongside time-invariant contributions from D_0_ states formed at the 2*h*ν_uv_ level. This is clearly manifested by the complementary transient signals of *m*/*z* 91 and 92, shown in Figure 10b, which suggest that the increase in the *m*/*z* 92 signal occurs at the expense of the *m*/*z* 91 signal. The transient recorded by gating both *m*/*z* 91 and 92 together, also shown in Figure 10b, elegantly demonstrates their complementary relationship. Accordingly, the additional rise and decay components of ∼2–3 ps observed in the fragment ion transients shown in Figure 10b can be ascribed to the lifetimes of DMPB^+^ excited states (D_n*_) populated by the UV pump pulse at the 3*h*ν_uv_ level. As discussed in Section 4.1, these lifetimes likely reflect rapid internal conversion to lower doublet states.

This interpretation is supported by the fragment ion transients measured with both pump and probe pulse energies reduced proportionally by a factor of 3, as shown in Figure 10d. At the significantly lower pump pulse energy (∼1.9 μJ/pulse), the fraction of cations produced at the 3*h*ν_uv_ level is greatly reduced. As a result, the fragment ions originate primarily from parent ions produced at the 2*h*ν_uv_ level, which are long-lived and give rise to time-invariant fragment ion transients.

Additional support comes from the branching ratio of *m*/*z* 91 to *m*/*z* 92 fragment ion production, denoted as the [91]/[92] ratio. Previous studies on alkylbenzene cations have shown that the [91]/[92] ratio is highly sensitive to the internal energy of the parent ion and typically increases with increasing internal energy.^83,85^ Assuming that ion fragmentation proceeds ergodically in the D_0_ state following internal conversion, this behavior suggests that barriers to *m*/*z* 91 ion production are higher than those to *m*/*z* 92 ion formation. In background mass spectra of DMPB recorded using only the ∼5.8 μJ/pulse UV pump pulse, the [91]/[92] ratio is about 0.75, which arises exclusively from D_n*_ states produced at the 3*h*ν_uv_ level. On the other hand, in the background-subtracted TRMS spectra of DMPB (see Figure 8b), the [91]/[92] ratio decreases from ∼0.4 at early delay times to ∼0.05 at about 10 ps, as shown in Figure 10c. It follows that in the nonbackground-subtracted TRMS, the [91]/[92] ratio decreases from ∼0.58 at early delay times to ∼0.4 at about 10 ps.

This behavior can be rationalized by recognizing that transient fragment ion signals originate from parent cations produced at the 2*h*ν_uv_ as well as the 3*h*ν_uv_ levels followed by absorption of a probe photon (*h*ν_vis_). At longer delay times (*t* > 10 ps), D_n*_-state populations produced at the 3*h*ν_uv_ level have decayed to minimal, and fragment ions originate exclusively from cationic D_*n*_ states reached at 2*h*ν_uv_ + *h*ν_vis_ level. These states lie lower in energy than the 3*h*ν_uv_ level, and therefore, resulting in a lower [91]/[92] ratio. On the other hand, at earlier times, fragment ions include contributions from the higher-lying states (D_n**_) at 3*h*ν_uv_ + *h*ν_vis_ level, giving rise to the larger [91]/[92] ratio due to the greater internal energy available prior to dissociation.

Thus, the interpretation described above accounts for the additional temporal components observed in fragment ion transients from DMPB^+^. In contrast, the *m*/*z* 91 fragment ion transients from PB^+^ show no such additional temporal features (see Figure 8a). This can be attributed to the fact that PB^+^ has only two low-energy dissociation pathways, both leading to fragment ions of the same mass (see Table 1 and Figure 14a). Pathways leading to the *m*/*z* 92 (C_7_H_8_^+^) fragment ion remain inactive even at energies above the 3*h*ν_uv_ level, as suggested by the TRMS spectra shown in Figure 8a. Therefore, although absorption of a probe photon may alter the branching ratio between the two pathways, the overall *m*/*z* 91 signal originating from PB^+^ remains unchanged, and the dynamics of D_n*_ excited states accessed at the 3*h*ν_uv_ level do not manifest in the fragment ion transients. On the other hand, the DMPB cation possesses four low-energy dissociation pathways that produce fragment ions of three different masses (see Table 1 and Figure 14b). As a result, the addition of a probe photon is more likely to affect the relative branching ratios among these channels, making the D_n*_ excited-state dynamics more readily observable in the fragment ion transients.

## 5 Conclusions

In this study, we investigated the photoionization-induced relaxation dynamics of two alkylbenzene cations, PB^+^ and DMPB^+^, with femtosecond PI–PF spectroscopy, using UV pump pulses well below the SFI regime to initiate photoionization via 1 + 1 REMPI and delayed probe pulses across the visible spectral region from 400 to 800 nm to induce photofragmentation of the evolving ionic systems. Our results and analyses reveal that, although the UV pump pulse used here induces substantial ionic fragmentation, only intact parent cations produced at the two-photon level contribute to the observed parent ion depletion transient signals. Highly excited cationic states initially accessed by absorption of additional UV photons within the pump pulse undergo nearly complete fragmentation due to their high internal energies, and therefore, do not contribute to the parent ion depletion transient signals. However, their dynamics may manifest in fragment ion formation transients, particularly when multiple competing dissociation pathways are involved.

TRPF spectra of PB^+^ and DMPB^+^ were obtained by measuring probe-induced parent ion depletion yield as a function of probe wavelength across the visible spectral region at a fixed delay time. These spectra are assigned to ionic resonance transitions with significant CT character, as suggested by TDDFT calculations. The pronounced polarization dependence of the parent ion depletion transients further supports the resonant excitation nature of the pump and probe transitions. Parent ion depletion transients of PB^+^ and DMPB^+^ exhibit a sub-100 fs ultrafast rise that can be attributed to initial vibrational dephasing and, in part, to resonant coherent excitation effects. Following this ultrafast initial rise, the parent ion depletion transients exhibit no temporal evolution over time scales exceeding 1 ns. This behavior is consistently observed at all probe wavelengths studied here, indicating that the associated TRPF spectra are time invariant.

The observed time invariance of the parent ion depletion signals indicates an absence of substantial relaxation following photoionization. This conclusion is consistent with common chemical intuition and supported by quantum chemical calculations, which predict that, upon photoionization, these alkylbenzene cations undergo only minimal conformational relaxation insufficient to alter their resonance absorption profile in the visible region. As such, alkylbenzene cations serve as ideal reference systems for interpreting ultrafast dynamics in more complex ionic systems that undergo reactions or substantial conformational relaxations following photoionization.

## Supplementary Material



## Notes

Additional experimental data, including all early time parent ion depletion transients, pulse energy dependences of parent ion depletion transient and TRMS; tabulated computational results, molecular orbitals associated with cationic resonance absorptions, Cartesian coordinates of optimized molecular structures (PDF)

## References

[1] Larsson M., Geppert W. D., Nyman G. (2012). Ion chemistry in space. Rep. Prog. Phys..

[2] Kobayashi K. (2019). Pulse Radiolysis
Studies for Mechanism in Biochemical Redox Reactions. Chem. Rev..

[3] Duncan M. A. (1997). Spectroscopy
of metal ion complexes: gas-phase model for solvation. Annu. Rev. Phys. Chem..

[4] Duncan M. A. (2000). Frontiers
in spectroscopy of mass-selected molecular ions. Int. J. Mass Spectrom..

[5] Duncan M. A. (2003). Infrared
spectroscopy to probe structure and dynamics in metal ion–molecule
complexes. Int. Rev. Phys. Chem..

[6] Bieske E., Maier J. (1993). Spectroscopic studies of ionic complexes
and clusters. Chem. Rev..

[7] Baer T., Dunbar R. C. (2010). Ion Spectroscopy: Where Did It Come From; Where Is
It Now; and Where Is It Going?. J. Am. Soc.
Mass Spectrom..

[8] Wolk A. B., Leavitt C. M., Garand E., Johnson M. A. (2014). Cryogenic Ion Chemistry
and Spectroscopy. Acc. Chem. Res..

[9] Bieske E. J., Dopfer O. (2000). High-Resolution Spectroscopy of Cluster Ions. Chem. Rev..

[10] Ebata T., Asuka F., Mikami N. (1998). Vibrational
spectroscopy of small-sized
hydrogen-bonded clusters and their ions. Int.
Rev. Phys. Chem..

[11] Zewail A. H. (2000). Femtochemistry:
Atomic-Scale Dynamics of the Chemical Bond. J. Phys. Chem. A.

[12] Dantus M., Zewail A. (2004). Introduction: Femtochemistry. Chem. Rev..

[13] Maiuri M., Garavelli M., Cerullo G. (2020). Ultrafast Spectroscopy: State of
the Art and Open Challenges. J. Am. Chem. Soc..

[14] Hertel I. V., Radloff W. (2006). Ultrafast dynamics in isolated molecules
and molecular
clusters. Rep. Prog. Phys..

[15] Nisoli M., Decleva P., Calegari F., Palacios A., Martín F. (2017). Attosecond
Electron Dynamics in Molecules. Chem. Rev..

[16] Ramasesha K., Leone S. R., Neumark D. M. (2016). Real-Time
Probing of Electron Dynamics
Using Attosecond Time-Resolved Spectroscopy. Annu. Rev. Phys. Chem..

[17] Martín F., Calegari F., Vozzi C., Ueda K., DiMauro L. (2024). Virtual Special
Issue on Attosecond Chemistry. J. Phys. Chem.
A.

[18] Ho J.-W., Chen W.-K., Cheng P.-Y. (2009). Femtosecond
pump-probe photoionization-photofragmentation
spectroscopy: Photoionization-induced twisting and coherent vibrational
motion of azobenzene cation. J. Chem. Phys..

[19] Shen C.-C., Tsai T.-T., Ho J.-W., Chen Y.-W., Cheng P.-Y. (2014). Communication:
Ultrafast time-resolved ion photofragmentation spectroscopy of photoionization-induced
proton transfer in phenol-ammonia complex. J.
Chem. Phys..

[20] Shen C.-C., Tsai T.-T., Wu J.-Y., Ho J.-W., Chen Y.-W., Cheng P.-Y. (2017). Watching proton transfer in real time: Ultrafast photoionization-induced
proton transfer in phenol-ammonia complex cation. J. Chem. Phys..

[21] Wu J.-Y., Cheng P.-Y. (2019). Ultrafast Protonation
of an Amide: Photoionization-Induced
Proton Transfer in Phenol-Dimethylformamide Complex Cation. J. Phys. Chem. A.

[22] Rosenberg M., Minitti M. P., Rusteika N., Bisgaard C. Z., Deb S., Weber P. M., Sølling T. I. (2010). Probing the Lifetimes of Internally
Excited Amyl Nitrite Cations. J. Phys. Chem.
A.

[23] Brogaard R. Y., Møller K. B., Sølling T. I. (2011). Real-Time Probing of Structural Dynamics
by Interaction between Chromophores. J. Phys.
Chem. A.

[24] Liu Y., Knopp G., Gerber T. (2014). Ultrafast
dynamics of ethylbenzene
cations probed by photofragmentation and photoelectron spectrometry. J. Mol. Struct..

[25] Liu Y., Gerber T., Zheng G., Xiao S., Cheng Q., Knopp G. (2017). Tracking ultrafast
dynamics of n-propylbenzene cations by delayed
photofragmentation and photoelectron spectroscopy. J. Mol. Spectrosc..

[26] Zuo W., Yin H., Liu X., Lv H., Zhao L., Shi Y., Yan B., Jin M., Ding D., Xu H. (2016). Identification of the
cationic excited state of cyclopentanone via time-resolved Ion yield
measurements. Chem. Phys. Lett..

[27] Geißler D., Pearson B. J., Weinacht T. (2007). Wave packet driven
dissociation and
concerted elimination in CH_2_I_2_. J. Chem. Phys..

[28] Bohinski T., Moore Tibbetts K., Tarazkar M., Romanov D., Matsika S., Levis R. J. (2013). Measurement
of an Electronic Resonance in a Ground-State,
Gas-Phase Acetophenone Cation via Strong-Field Mass Spectrometry. J. Phys. Chem. Lett..

[29] Bohinski T., Moore Tibbetts K., Tarazkar M., Romanov D. A., Matsika S., Levis R. J. (2014). Strong
Field Adiabatic Ionization Prepares a Launch
State for Coherent Control. J. Phys. Chem. Lett..

[30] Munkerup K., Romanov D., Bohinski T., Stephansen A. B., Levis R. J., Sølling T. I. (2017). Conserving Coherence and Storing
Energy during Internal Conversion: Photoinduced Dynamics of cis- and
trans-Azobenzene Radical Cations. J. Phys. Chem.
A.

[31] Word M. K. D., López Peña H. A., Ampadu Boateng D., McPherson S. L., Gutsev G. L., Gutsev L. G., Lao K. U., Tibbetts K. M. (2022). Ultrafast Dynamics of Nitro-Nitrite
Rearrangement and
Dissociation in Nitromethane Cation. J. Phys.
Chem. A.

[32] López
Peña H. A., Shusterman J. M., Dalkiewicz C., McPherson S. L., Dunstan C., Sangroula K., Lao K. U., Tibbetts K. M. (2024). Photodissociation Dynamics of the
Highly Stable ortho-Nitroaniline Cation. J.
Phys. Chem. A.

[33] Britt E., López Peña H. A., Shusterman J. M., Sangroula K., Lao K. U., Tibbetts K. M. (2025). Ultrafast
Dissociation
Dynamics of the Sensitive Explosive Ethylene Glycol Dinitrate. J. Phys. Chem. Lett..

[34] Konar A., Shu Y., Lozovoy V. V., Jackson J. E., Levine B. G., Dantus M. (2014). Polyatomic
Molecules under Intense Femtosecond Laser Irradiation. J. Phys. Chem. A.

[35] Dantus M. (2024). Tracking Molecular
Fragmentation in Electron-Ionization Mass Spectrometry with Ultrafast
Time Resolution. Acc. Chem. Res..

[36] Li S., Jochim B., Jackson J. E., Dantus M. (2021). Femtosecond dynamics
and coherence of ionic retro-Diels-Alder reactions. J. Chem. Phys..

[37] Stamm J., Kwon S., Sandhu S., Shaik M., Das R., Sandhu J., Curenton B., Wicka C., Levine B. G., Sun L. (2023). The Surprising
Dynamics of the McLafferty Rearrangement. J.
Phys. Chem. Lett..

[38] Stamm J., Li S., Jochim B., Yuwono S. H., Priyadarsini S. S., Piecuch P., Dantus M. (2022). Femtosecond
intramolecular rearrangement
of the CH_3_NCS radical cation. J.
Chem. Phys..

[39] Kwon S., Stamm J., Dantus M. (2025). Ultrafast Dynamics and Rearrangement
of the EUV Photoacid Generator Phenyl Triflate. J. Phys. Chem. Lett..

[40] Zhou L., Liu Y., Sun T., Feng S., Lv H., Xu H. (2019). Ultrafast
Evolution of B^2^E_2g_ - X^2^E_1g_ Conical Intersection of Benzene Cations by Strong Field Ionization-Photo
Fragmentation. J. Phys. Chem. A.

[41] Zhou L., Liu Y., Sun T., Yin H., Zhao Y., Lv H., Xu H. (2021). Strong Field Ionization-Photofragmentation
on Ultrafast Evolution
of Electronic States of Toluene Cations. J.
Phys. Chem. A.

[42] Zhou L.-X., Liu Y., He S., Gao D.-S., Shen X.-C., Chen Q., Yu T., Lv H., Xu H.-F. (2022). Ultrafast dynamics of cationic electronic
states of vinyl bromide by strong-field ionization-photofragmentation. Chin. Phys. B.

[43] Bhattacharyya S., Wang E., Borne K., Chen K., Venkatachalam A. S., Lam H. V. S., Ziaee F., Pathak S., Khmelnitskiy A., Carnes K. D. (2024). Delayed
Dissociation and Transient Isomerization
during the Ultrafast Photodissociation of the Tribromomethane Cation. J. Phys. Chem. Lett..

[44] Tanaka M., Kawaji M., Yatsuhashi T., Nakashima N. (2009). Ionization
and Fragmentation of Alkylphenols by 0.8–1.5 μm Femtosecond
Laser Pulses. J. Phys. Chem. A.

[45] Weinkauf R., Aicher P., Wesley G., Grotemeyer J., Schlag E. W. (1994). Femtosecond versus Nanosecond Multiphoton Ionization
and Dissociation of Large Molecules. J. Phys.
Chem..

[46] Dietz W., Neusser H. J., Boesl U., Schlag E. W., Lin S. H. (1982). A Model
for Multiphoton Ionisation Mass Spectroscopy with Application to Benzene. Chem. Phys..

[47] Schlag E. W., Neusser H. J. (1983). Multiphoton mass
spectrometry. Acc. Chem. Res..

[48] Boesl U. (1991). Multiphoton
excitation and mass-selective ion detection for neutral and ion spectroscopy. J. Phys. Chem..

[49] DeWitt M. J., Levis R. J. (1995). Near-infrared femtosecond photoionization/dissociation
of cyclic aromatic hydrocarbons. J. Chem. Phys..

[50] DeWitt M. J., Levis R. J. (1998). Observing the Transition from a Multiphoton-Dominated
to a Field-Mediated Ionization Process for Polyatomic Molecules in
Intense Laser Fields. Phys. Rev. Lett..

[51] Lezius M., Blanchet V., Ivanov M. Y., Stolow A. (2002). Polyatomic molecules
in strong laser fields: Nonadiabatic multielectron dynamics. J. Chem. Phys..

[52] Keldysh L.
V. (1965). Ionization
in the Field of a Strong Electromagnetic Wave. J. Exp. Theor. Phys..

[53] Levis R. J., DeWitt M. J. (1999). Photoexcitation,
Ionization, and Dissociation of Molecules
Using Intense Near-Infrared Radiation of Femtosecond Duration. J. Phys. Chem. A.

[54] Ledingham K. W. D., Singhal R. P., Smith D. J., McCanny T., Graham P., Kilic H. S., Peng W. X., Wang S. L., Langley A. J., Taday P. F. (1998). Behavior of Polyatomic Molecules in Intense
Infrared Laser Beams. J. Phys. Chem. A.

[55] Yatsuhashi T., Nakashima N. (2005). Effects of Polarization of 1.4 μm Femtosecond
Laser Pulses on the Formation and Fragmentation of Naphthalene Molecular
Ions Compared at the Same Effective Ionization Intensity. J. Phys. Chem. A.

[56] Tanaka M., Panja S., Murakami M., Yatsuhashi T., Nakashima N. (2006). Intact molecular ion formation of
cyclohexane and 2,3-dimethyl-1,3-butadiene
by excitation with a short, intense femtosecond laser pulse. Chem. Phys. Lett..

[57] Harada H., Shimizu S., Yatsuhashi T., Sakabe S., Izawa Y., Nakashima N. (2001). A key factor
in parent and fragment ion formation on
irradiation with an intense femtosecond laser pulse. Chem. Phys. Lett..

[58] Murakami M., Mizoguchi R., Shimada Y., Yatsuhashi T., Nakashima N. (2005). Ionization
and fragmentation of anthracene with an
intense femtosecond laser pulse at 1.4μm. Chem. Phys. Lett..

[59] Zhang J.-F., Lü H., Zuo W.-L., Xu H.-F., Jin M.-X., Ding D.-J. (2015). Ionizations
and fragmentations of benzene, methylbenzene,
and chlorobenzene in strong IR and UV laser fields. Chin. Phys. B.

[60] Smith S. M., Li X., Markevitch A., Romanov D., Levis R. J., Schlegel H. B. (2007). Numerical
Simulation of Nonadiabatic Electron Excitation in the Strong-Field
Regime. 3. Polyacene Neutrals and Cations. J.
Phys. Chem. A.

[61] Chu X., Chu S.-I. (2004). Role of the electronic
structure and multielectron
responses in ionization mechanisms of diatomic molecules in intense
short-pulse lasers: An all-electron ab initio study. Phys. Rev. A.

[62] Yahel E., Natan A. (2018). Effect of multiorbital
contributions to strong-field ionization of
benzene derivatives. Phys. Rev. A.

[63] Boguslavskiy A. E., Mikosch J., Gijsbertsen A., Spanner M., Patchkovskii S., Gador N., Vrakking M. J. J., Stolow A. (2012). The Multielectron Ionization
Dynamics Underlying Attosecond Strong-Field Spectroscopies. Science.

[64] Akagi H., Otobe T., Staudte A., Shiner A., Turner F., Dörner R., Villeneuve D. M., Corkum P. B. (2009). Laser Tunnel Ionization
from Multiple Orbitals in HCl. Science.

[65] Fukahori S., Kubo A., Hasegawa H. (2024). Dissociative ionization
and post-ionization
alignment of aligned O_2_ in an intense femtosecond laser
field. Phys. Chem. Chem. Phys..

[66] Sándor P., Zhao A., Rozgonyi T., Weinacht T. (2014). Strong field molecular
ionization to multiple ionic states: direct versus indirect pathways. J. Phys. B: At., Mol. Opt. Phys..

[67] Zhao A., Sándor P., Rozgonyi T., Weinacht T. (2014). Removing electrons
from more than one orbital: direct and indirect pathways to excited
states of molecular cations. J. Phys. B: At.,
Mol. Opt. Phys..

[68] Lin M.-F., Neumark D. M., Gessner O., Leone S. R. (2014). Ionization and dissociation
dynamics of vinyl bromide probed by femtosecond extreme ultraviolet
transient absorption spectroscopy. J. Chem.
Phys..

[69] Allum F., McManus J., Denby O., Burt M., Brouard M. (2022). Photoionization
and Photofragmentation Dynamics of I_2_ in Intense Laser
Fields: A Velocity-Map Imaging Study. J. Phys.
Chem. A.

[70] Liu H., Zhao S.-F., Li M., Deng Y., Wu C., Zhou X.-X., Gong Q., Liu Y. (2013). Molecular-frame photoelectron
angular distributions of strong-field tunneling from inner orbitals. Phys. Rev. A.

[71] Corkum P. B. (1993). Plasma
perspective on strong field multiphoton ionization. Phys. Rev. Lett..

[72] Tagliamonti V., Sándor P., Zhao A., Rozgonyi T., Marquetand P., Weinacht T. (2016). Nonadiabatic dynamics and multiphoton resonances in
strong-field molecular ionization with few-cycle laser pulses. Phys. Rev. A.

[73] In this study, the UV (λ = 266.4 nm) beam is focused by a *f* = 300 mm lens with an input beam diameter of ∼ 3 mm. Microscopic inspection of burned spots at the focal plane on a thin metal foil revealed a focal spot diameter of ∼ 50 μm, which is consistent with a beam quality parameter of M^2^≈1.5. Given the pulse widths (fwhm) of ∼ 120 fs, the peak laser irradiance at the focus is estimated to be ∼ 4 × 10^11^ W cm^–2^ for a pulse energy of 1 μJ/pulse.

[74] Takahashi M., Kimura K. (1992). Cation vibrational spectroscopy of
trans and gauche n-propylbenzene
rotational isomers. Two-color threshold photoelectron study and ab initio
calculations. J. Chem. Phys..

[75] Weinkauf R., Lehrer F., Schlag E. W., Metsala A. (2000). Investigation of charge
localization and charge delocalization in model molecules by multiphoton
ionization photoelectron spectroscopy and DFT calculations. Faraday Discuss..

[76] Seeman J. I., Secor H. V., Breen P. J., Grassian V. H., Bernstein E. R. (1989). A study
of nonrigid aromatic molecules. Observation and spectroscopic analysis
of the stable conformations of various alkylbenzenes by supersonic
molecular jet laser spectroscopy. J. Am. Chem.
Soc..

[77] Bischof P. K., Dewar M. J. S., Goodman D. W., Jones T. B. (1974). Photoelectron spectra
of molecules: VI. Hyperconjugation versus *p*
_π_-*d*
_π_ bonding in group IVb compounds. J. Organomet. Chem..

[78] Lias, S. G. ; Bartmess, J. E. ; Liebman, J. F. ; Holmes, J. L. ; Levin, R. D. ; Mallard, W. G. Gas-Phase Ion and Neutral Thermochemistry. J. Phys. Chem. Ref. Data 1988, 17.

[79] Ruscic, B. ; Bross, D. H. Active Thermochemical Tables (ATcT) Thermochemical Values ver. 1.202; Argonne National Laboratory (ANL): Argonne, IL, USA, 2024.

[80] Kuck D. (1990). Mass spectrometry
of alkylbenzenes and related compounds. Part I. Gas-phase ion chemistry
of alkylbenzene radical cations. Mass Spectrom.
Rev..

[81] Smith, R. M. , Important Mass Spectral Rearrangements. In Understanding Mass Spectra: A Basic Approach, 2nd ed.; Wiley-Interscience, 2004; pp 207–237.

[82] Gross, J. Mass Spectrometry: a Textbook, 3rd ed., Chapter 6; Springer, 2017.

[83] Halbert S., Bouchoux G. (2012). Isomerization and Dissociation of n-Butylbenzene Radical
Cation. J. Phys. Chem. A.

[84] The natural abundance ion signal ratio ([M + 1]/[M]) for the C_7_H_7_ ^+^ ion is about 8.1%, while the measured [92]/[91] ratio is 10.5 ± 0.6% in the PB mass spectra recorded using UV pulse energy of 1–10 μJ/pulse.

[85] Hwang W. G., Moon J. H., Choe J. C., Kim M. S. (1998). Dissociation Dynamics
of n-Propylbenzene Molecular Ion. J. Phys. Chem.
A.

[86] NIST Chemistry WebBook, NIST Standard Reference Database Number 69. https://webbook.nist.gov/chemistry/.

[87] Nathanson G. M., McClelland G. M. (1984). Fluorescence
polarization as a probe of the rotational
dynamics of isolated highly excited molecules. J. Chem. Phys..

[88] Baskin J. S., Felker P. M., Zewail A. H. (1987). Purely
Rotational Coherence Effect
and Time-Resolved Sub-Doppler Spectroscopy of Large Molecules.2. Experimental. J. Chem. Phys..

[89] Baskin J.
S., Zewail A. H. (1994). Femtosecond
Real-Time Probing of Reactions.15. Time-Dependent
Coherent Alignment. J. Phys. Chem..

[90] Baskin J. S., Banares L., Pedersen S., Zewail A. H. (1996). Femtosecond
real-time
probing of reactions.20. Dynamics of twisting, alignment, and IVR
in the trans-stilbene isomerization reaction. J. Phys. Chem..

[91] Anand R., LeClaire J. E., Johnson P. M. (1999). Photoinduced
Rydberg Ionization (PIRI)
Spectroscopy of the B̃ State of the Fluorobenzene Cation. J. Phys. Chem. A.

[92] Walter K., Boesl U., Schlag E. W. (1989). Molecular
ion spectroscopy: Resonance-enhanced
multiphoton dissociation spectra of the fluorobenzene cation. Chem. Phys. Lett..

[93] A small discrepancy (<10%) is present and is likely due to the difference in collection and/or detection efficiency for the parent and fragment ions.

[94] Since the cations are produced with an average vibrational energy of ∼ 0.23 eV, the effective dissociation limits are likely lowered by approximately the same amount.

[95] Dunbar R. C. (1979). Photodissociation
spectroscopy of alkylbenzene cations. J. Phys.
Chem..

[96] Dymerski P. P., Fu E., Dunbar R. C. (1974). Ion cyclotron
resonance photodissociation spectroscopy
spectra of substituted benzenes. J. Am. Chem.
Soc..

[97] Frisch, M. J. ; Trucks, G. W. ; Schlegel, H. B. ; Scuseria, G. E. ; Robb, M. A. ; Cheeseman, J. R. ; Scalmani, G. ; Barone, V. ; Petersson, G. A. ; Nakatsuji, H. ; Gaussian 16, Revision B.01; Gaussian, Inc.: Wallingford CT, 2016.

[98] Due to its symmetry, the *gauche*-PB conformer has a statistical weight of two, which can compensate for its slightly higher energy.

[99] Lehrer F., Weinkauf R., Metsala A. (2007). Comparison of photoelectron-spectroscopy
results to ab-initio and density functional calculations: The ethylbenzene
cation. Z. Physiol. Chem..

[100] Nagy-Felsobuki F., Peel J. (1979). Photoelectron spectroscopic
studies
of the butylbenzenes. J. Electron Spectrosc.
Relat. Phenom..

[101] Kim B., Weber P. M. (1995). Two-Color Laser
Photoelectron Spectroscopy of Electronically
Excited Cations: Vibrationally Resolved Spectrum of the ^2^A_2_ State of Aniline^+^. J. Phys. Chem..

[102] Goode J. G., LeClaire I. E., Johnson P. M. (1996). Probing
the photoinduced
Rydberg ionization process. Int. J. Mass Spectrom.
Ion Processes.

[103] Johnson P. M., Anand R., Hofstein J. D., LeClaire J. E. (2000). Reassessing
the orbitals of pi systems using photoinduced Ryberg ionization spectroscopy. J. Electron Spectrosc. Relat. Phenom..

[104] Schick C. P., Weber P. M. (2001). Ultrafast dynamics in superexcited
states of phenol. J. Phys. Chem. A.

[105] Zhou Z., Xie M., Wang Z., Qi F. (2009). Determination
of absolute photoionization cross-sections of aromatics and aromatic
derivatives. Rapid Commun. Mass Spectrom..

[106] Gallmann L., Jordan I., Wörner H. J., Castiglioni L., Hengsberger M., Osterwalder J., Arrell C. A., Chergui M., Liberatore E., Rothlisberger U. (2017). Photoemission and photoionization time delays
and rates. Struct. Dyn..

[107] Huppert M., Jordan I., Baykusheva D., von Conta A., Wörner H. J. (2016). Attosecond Delays in Molecular Photoionization. Phys. Rev. Lett..

[108] Tong J., Liu X., Dong W., Jiang W., Zhu M., Xu Y., Zuo Z., Lu P., Gong X., Song X. (2022). Probing Resonant Photoionization
Time Delay by Self-Referenced
Molecular Attoclock. Phys. Rev. Lett..

[109] Boyer A., Loriot V., Nandi S., Lépine F. (2024). Probing Photoionization
Dynamics in Acetylene with Angle-Resolved Attosecond Interferometry. J. Phys. Chem. A.

[110] Li X., Liu Y., Zhang D., He L., Luo S., Shu C.-C., Ding D. (2023). Visualizing vibrationally
resolved
attosecond time delay in resonance-enhanced multiphoton ionization
of NO molecules. Phys. Rev. A.

[111] Köhnke D., Bayer T., Wollenhaupt M. (2024). Measurement
of ionization delays in atomic resonance-enhanced multiphoton ionization
using photoelectron vortices. Phys. Rev. A.

[112] Callegari A., Srivastava H. K., Merker U., Lehmann K. K., Scoles G., Davis M. J. (1997). Eigenstate resolved infrared-infrared
double-resonance study of intramolecular vibrational relaxation in
benzene: First overtone of the CH stretch. J.
Chem. Phys..

[113] Callegari A., Merker U., Engels P., Srivastava H. K., Lehmann K. K., Scoles G. (2000). Intramolecular vibrational
redistribution
in aromatic molecules. I. Eigenstate resolved CH stretch first overtone
spectra of benzene. J. Chem. Phys..

[114] Aßmann J., Kling M., Abel B. (2003). Watching Photoinduced
Chemistry and Molecular Energy Flow in Solution in Real Time. Angew. Chem., Int. Ed..

[115] Gruebele M., Bigwood R. (1998). Molecular vibrational energy flow:
Beyond the Golden Rule. Int. Rev. Phys. Chem..

[116] Nesbitt D. J., Field R. W. (1996). Vibrational Energy Flow in Highly
Excited Molecules: Role of Intramolecular Vibrational Redistribution. J. Phys. Chem..

[117] The density of vibrational states were calculated using the Beyer–Swinehart direct state-counting algorithm for harmonic oscillators with vibrational frequencies obtained in CBS-QB3 calculations.

[118] Opposite trends may occur on the red or blue wings of spectra; however, such effects are difficult to observe due to low depletion yields in these regions.

[119] Lee I. R., Chen W.-K., Chung Y.-C., Cheng P.-Y. (2000). A Direct
Observation of Non-RRKM Behavior in Femtosecond Photophysically Activated
Reactions. J. Phys. Chem. A.

[120] Lee I.-R., Chung Y.-C., Chen W.-K., Hong X.-P., Cheng P.-Y. (2001). Femtosecond
probing of photodissociation dynamics in
acyl cyanides. J. Chem. Phys..

[121] Conde A. P., Montero R., Longarte A. (2021). Influence of coherent
adiabatic excitation on femtosecond transient signals. Phys. Scr..

