# Supplementary material for: Femtosecond Time- and Spectrally Resolved Ion Photofragmentation Spectroscopy: Case Studies of Two Alkylbenzene Cations
Source: J Phys Chem A. 2025 Jul 21;129(30):6845–65. doi: 10.1021/acs.jpca.5c04149 (PMC12319915; doi:10.1021/acs.jpca.5c04149)
Supplement: Supplementary file 1 [file jp5c04149_si_001.pdf]

Supporting Information

Femtosecond Time- and Spectrally Resolved Ion Photofragmentation Spectroscopy: Case Studies of Two Alkylbenzene Cations

Chen-Yi Chu,[#] Hsin Liu,[#] and Po-Yuan Cheng*

Department of Chemistry, National Tsing Hua University, Hsinchu 300044, Taiwan, Republic of China

Author Contributions

[#]C.C. and H.L. contributed equally to this work

*Corresponding author, Email: pycheng@mx.nthu.edu.tw

S1. Supporting Information Figures and Tables

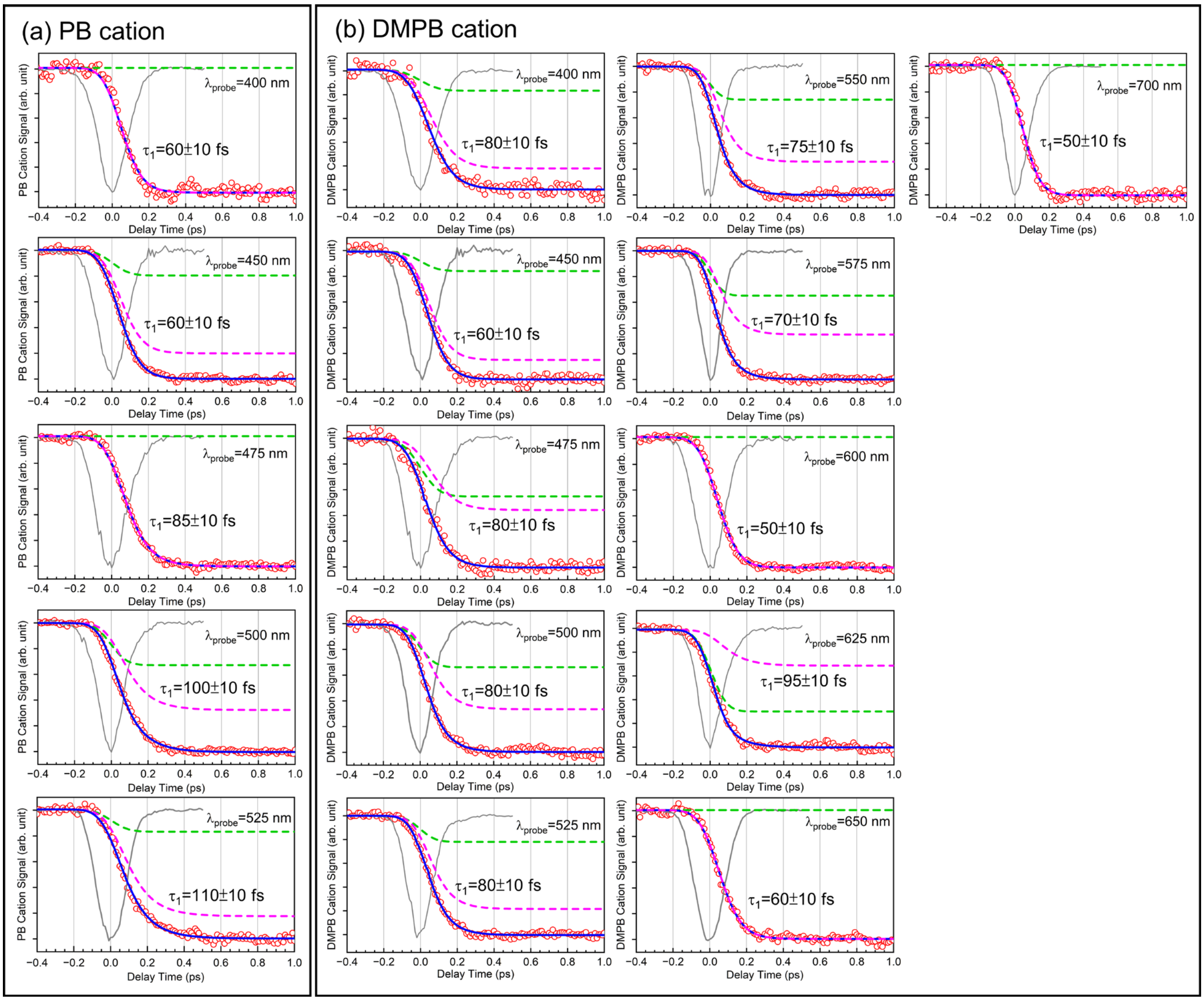

Figure S1. Early-time parent ion depletion transients of (a) PB and (b) DMPB cations, measured at various probe wavelengths, including those shown in Figure 7. Also shown are the best fits using the two-component model function described in the main text. Probe wavelengths are indicated in each panel. Experimental data are shown as red open circles, and the best-fit curves are displayed as blue solid lines. Colored dashed lines represent the decomposed components of the fit: (green) an instantaneous rise for parent cations with low vibrational excitation, and (pink) an exponential rise for parent cations with high vibrational excitation. Extracted rise times (τ_1) from the best fits are also provided for each case.

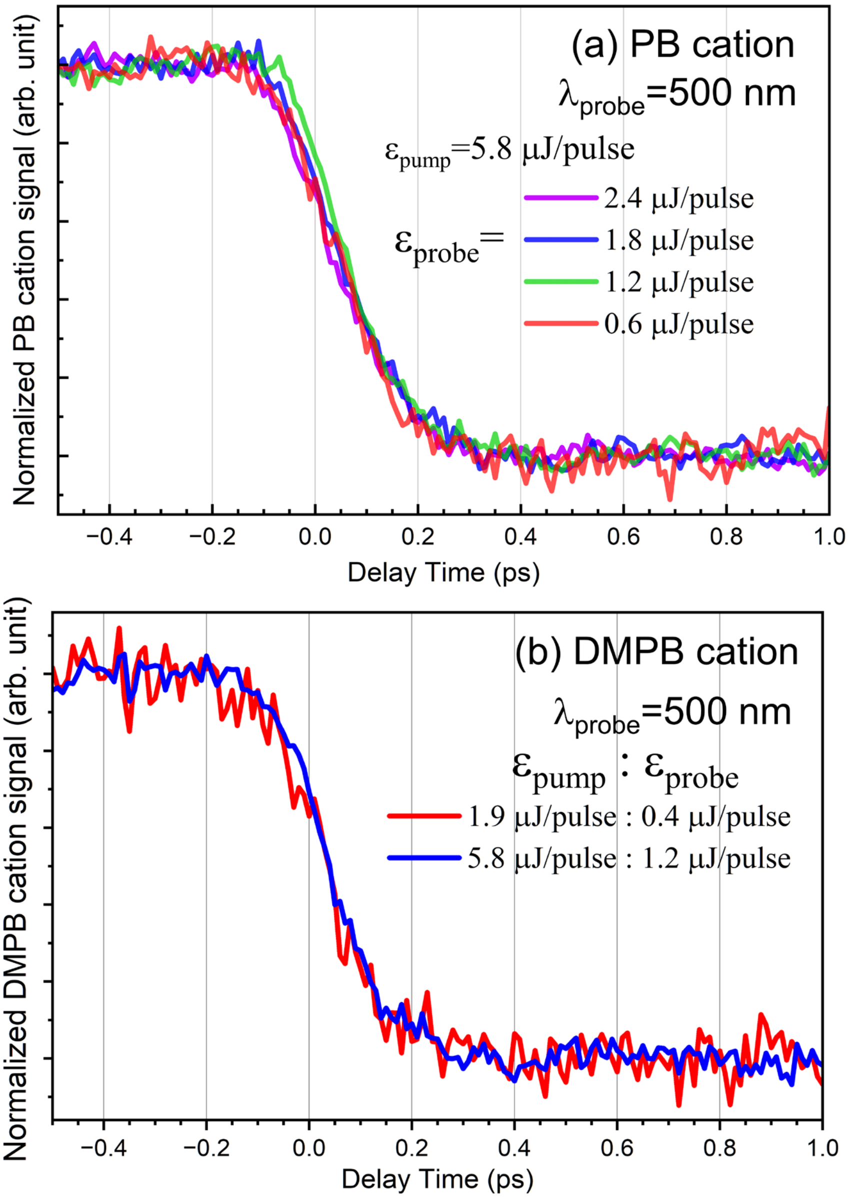

Figure S2. (a) Probe pulse energy dependence of early-time PB parent ion depletion transients measured at four probe pulse energies (ε_{probe}) ranging from 0.6 to 2.4 μJ/pulse, with the pump pulse energy (ε_{pump}) held constant. (b) Early-time DMPB parent ion depletion transients measured with both pump and probe pulse energies reduced by a factor of three (red trace) from the typical laser condition (blue trace). In all cases, the depletion signal reaches its half-maximum level at positive delay times of several tens of femtoseconds, suggesting that the delayed onset is not caused by saturation effects or nonlinear enhancement of ionization at time zero.

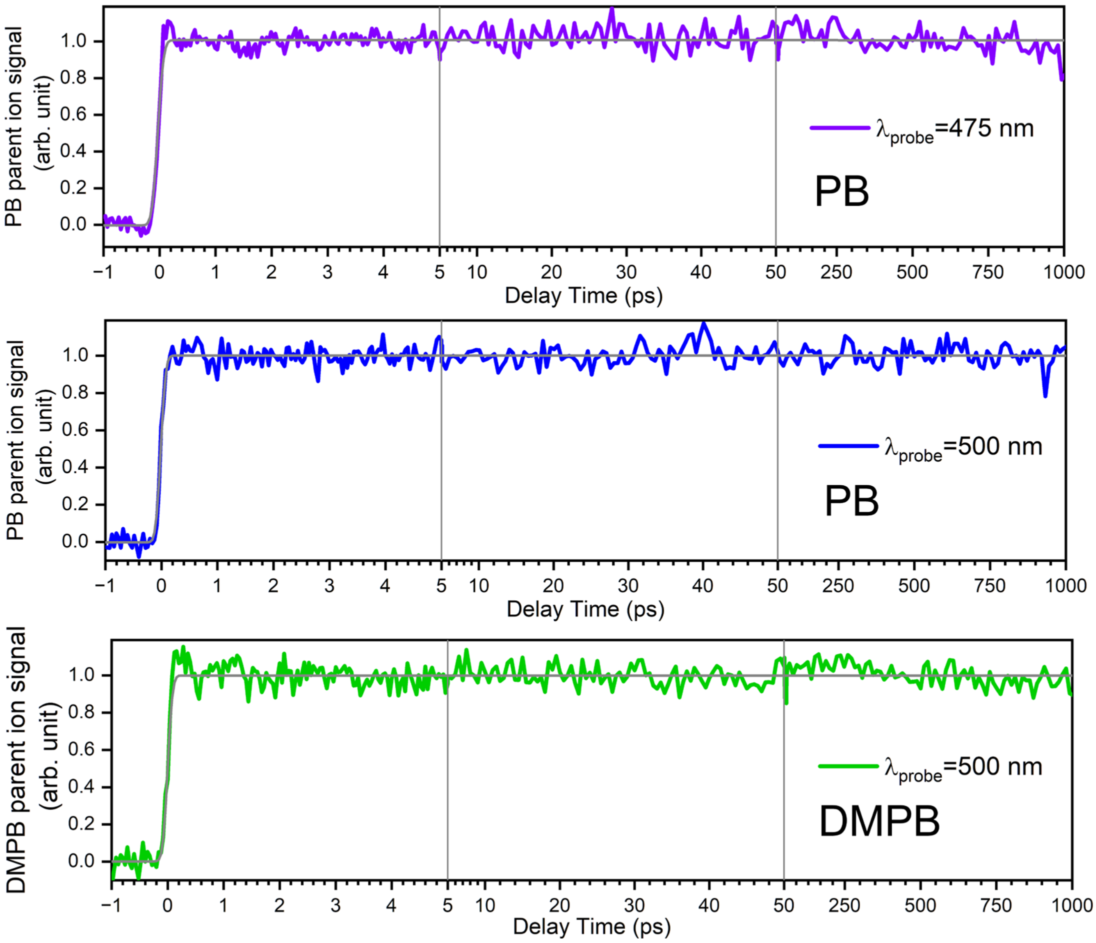

Figure S3. PE–MPI parent ion enhancement transients of PB and DMPB measured at selected probe wavelengths using a reduced pump pulse energy (<0.05 μJ/pulse) and an elevated probe pulse energy (~5-6 μJ/pulse). Under these conditions, no parent ion signal was detectable with the pump pulse alone. All other experimental conditions are identical to those used for the ion depletion transients shown in Figure 6. As described in the main text, these PE–MPI transients reflect the dynamics of the neutral S_1 state excited by the UV pump pulse. The absence of any noticeable temporal evolution of these transients within one nanosecond indicates the long-lived nature of the neutral alkylbenzene S_1 states near their origin, which typically have lifetimes much longer than the time window probed in this study.

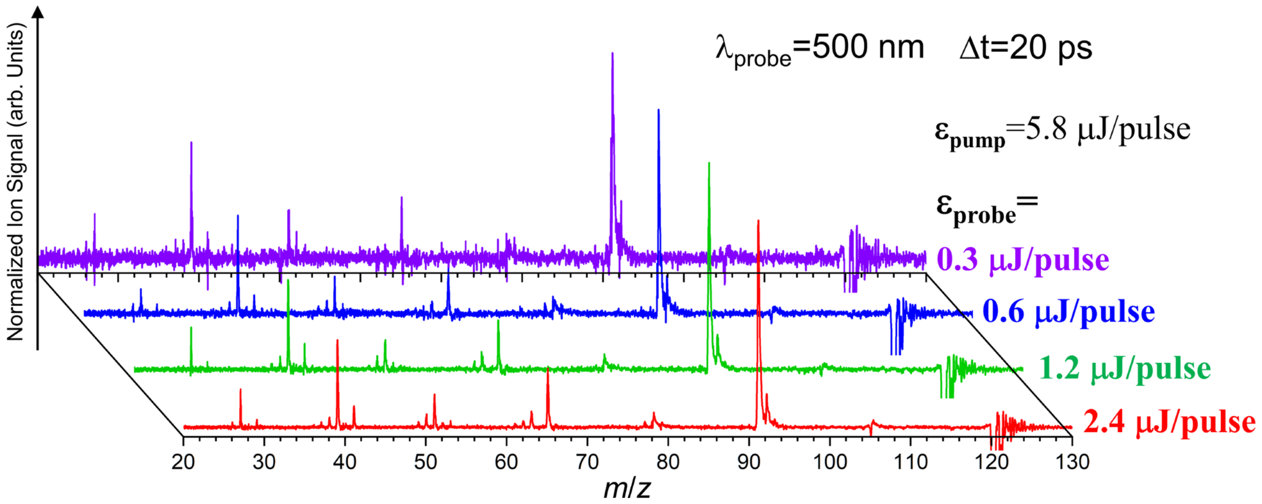

Figure S4. Background-subtracted TRMS spectra of PB cation measured at λ_{probe}= 500 nm and Δt=20 ps with probe pulse energies varied over an eightfold range from 0.3 to 2.4 μJ/pulse, while the UV pump pulse was kept constant at 5.8 μJ/pulse. For clarity, the parent ion depletion signal has been truncated, and each spectrum is normalized to its most intense fragment-ion peak. The relative intensity pattern of lighter fragment ions (m/z < 80) compared to the major fragment ion at m/z 91 remains essentially unchanged, indicating that their formation does not result from two-photon absorption of the probe pulse.

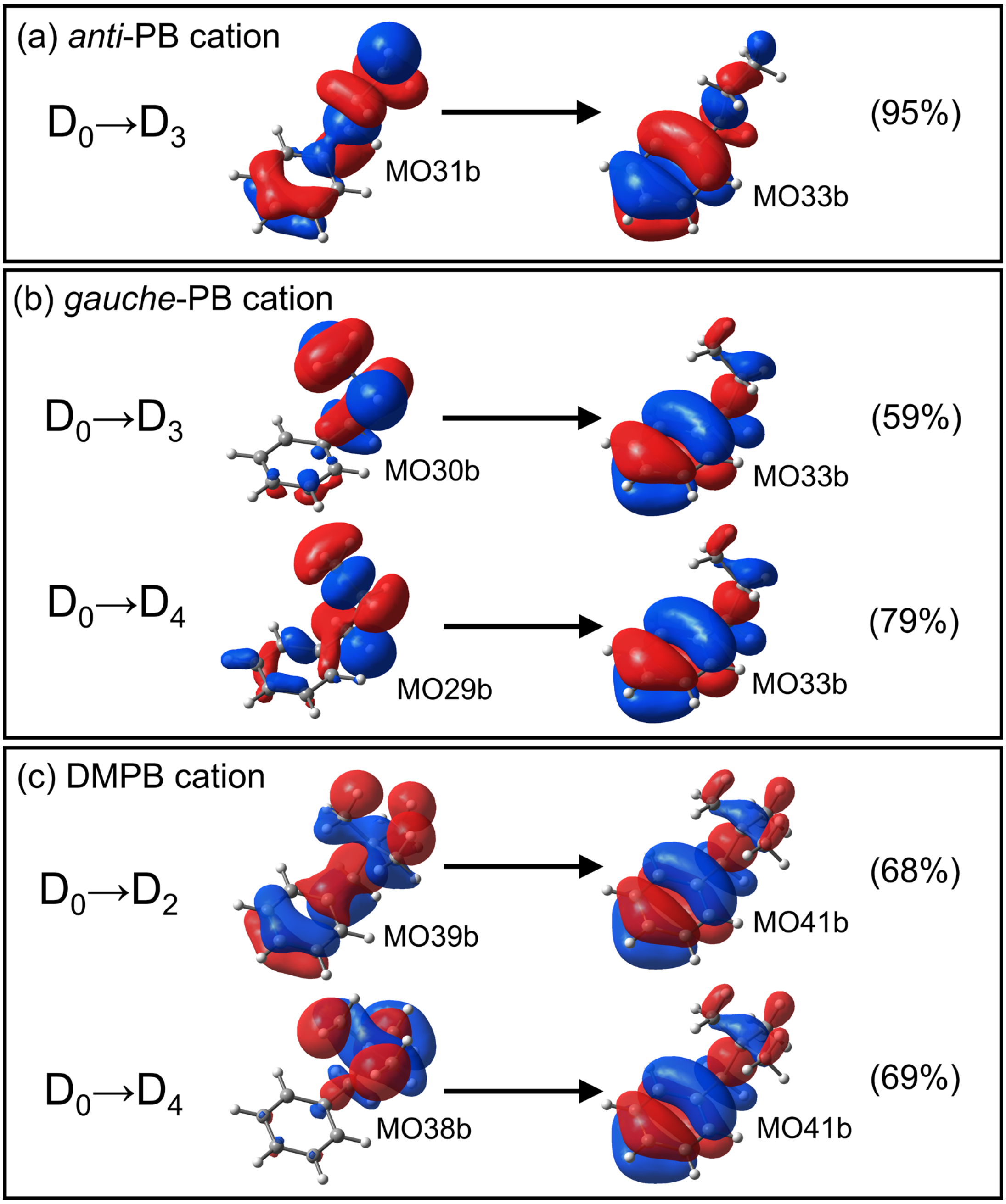

Figure S5. Dominant molecular orbital (MO) excitations contributing to the major resonance transitions of PB and DMPB cations in the visible spectral region, calculated at the TD-B3LYP/cc-pVTZ level at optimized geometries of cationic ground states. The percentage in parentheses indicates the contribution of each MO excitation to the corresponding resonance transition.

Table S1. Calculated relative energies (in eV) of cationic states at the neutral ground-state optimized structure, referenced to the neutral ground-state minimum

	anti-PB		*gauche*-PB		DMPB	
	ΔE[a] at S_0 min.	osc.[b]	ΔE[a] at S_0 min.	osc.[b]	ΔE[a] at S_0 min.	osc.[b]
S_0 min.	0		0		0	
D_0	9.024	N.A.	9.036	N.A.	8.963	N.A.
D_1	9.408	0.0000	9.411	0.0000	9.367	0.0000
D_2	10.617	0.0000	10.665	0.0001	10.264	0.0001
D_3	10.962	0.0024	10.808	0.0021	10.32	0.0000
D_4	11.056	0.0668	11.185	0.0632	10.807	0.1037
D_5	11.611	0.0000	11.669	0.0029	11.319	0.0004
D_6	12.016	0.0647	11.831	0.0002	11.401	0.0002
D_7	12.149	0.0000	12.184	0.0259	11.462	0.0010
D_8	12.372	0.0046	12.485	0.0000	11.856	0.0006
D_9	13.041	0.0149	13.14	0.0087	12.025	0.0236
D_{10}	13.382	0.0153	13.347	0.0025	12.232	0.0054
D_{11}	13.772	0.0000	13.673	0.0119	12.353	0.0000
D_{12}	13.774	0.0078	13.851	0.0110	12.953	0.0032
D_{13}	13.848	0.0106	13.9	0.0036	13.256	0.0039
D_{14}	13.981	0.0002	14.118	0.0288	13.659	0.0024
D_{15}	14.552	0.0000	14.558	0.0162	13.757	0.0089
D_{16}	14.599	0.0971	14.62	0.0597	13.793	0.0032
D_{17}	14.736	0.0006	14.749	0.0010	13.851	0.0003
D_{18}	15.094	0.0000	15.028	0.0009	14.005	0.0465
D_{19}	15.106	0.0003	15.122	0.0002	14.419	0.0849
D_{20}	15.456	0.0019	15.286	0.0044	14.431	0.0000

[a]Relative energies (in eV) of cationic states referenced to the neutral ground-state minimum. The relative energy is calculated as $\Delta E = VIE + VEE^+(S_0 \text{ min})$, where VIE is the vertical ionization energy calculated at the CBS-QB3 level, and $VEE^+(S_0 \text{ min})$ is the vertical excitation energy of the cation calculated at the TD-B3LYP/cc-pVTZ level at the optimized neutral ground-state structure.
[b]Oscillator strengths for cationic $D_0 \rightarrow D_n$ transitions calculated at the TD-B3LYP/cc-pVTZ level of theory at the neutral ground state optimized structure. Excited states near the three-UV-photon energy region (~13.35–13.95 eV) with appreciable oscillator strengths suggest efficient post-ionization resonance absorptions of a third UV photon.

S2. Neutral and Cationic ground-state optimized structures calculated at CBS-QB3 and ground to excited state transition electric dipole moments calculated at TD-B3LYP/cc-pVTZ

(1) neutral *anti*-PB ground state

Cartesian coordinates

	X	Y	Z
C	-0.3898470000	-2.1425370000	1.2034080000
C	-0.3898470000	-0.7495770000	1.2003510000
C	-0.3881530000	-0.0306490000	0.0000000000
C	-0.3898470000	-0.7495770000	-1.2003510000
C	-0.3898470000	-2.1425370000	-1.2034080000
C	-0.3893130000	-2.8446050000	0.0000000000
H	-0.3952290000	-2.6798660000	2.1456500000
H	-0.3966580000	-0.2109140000	2.1432200000
H	-0.3966580000	-0.2109140000	-2.1432200000
H	-0.3952290000	-2.6798660000	-2.1456500000
H	-0.3927830000	-3.9289170000	0.0000000000
C	-0.3467470000	1.4811080000	0.0000000000
H	-0.8808330000	1.8622320000	-0.8778000000
H	-0.8808330000	1.8622320000	0.8778000000
C	1.0853260000	2.0504150000	0.0000000000
H	1.6208770000	1.6669950000	0.8755460000
H	1.6208770000	1.6669950000	-0.8755460000
C	1.1182240000	3.5811570000	0.0000000000
H	0.6157020000	3.9883720000	0.8831180000
H	2.1453610000	3.9561000000	0.0000000000
H	0.6157020000	3.9883720000	-0.8831180000

Ground to excited state transition electric dipole moments (Au):

state	X	Y	Z	Dip. S.	Osc.
1	-0.0000	-0.0000	0.1121	0.0126	0.0016

(2) neutral *gauche*-PB ground state

Cartesian coordinates

	X	Y	Z
C	1.9071540000	1.2567910000	0.1024120000
C	0.6042520000	1.0760760000	0.5643510000
C	-0.0099330000	-0.1794310000	0.5196330000
C	0.7233820000	-1.2541270000	0.0012550000

C	2.0245720000	-1.0796650000	-0.4614170000
C	2.6218950000	0.1790960000	-0.4134500000
H	2.3637220000	2.2395030000	0.1489290000
H	0.0570930000	1.9212730000	0.9697330000
H	0.2706950000	-2.2405240000	-0.0334000000
H	2.5753560000	-1.9272690000	-0.8546180000
H	3.6362790000	0.3163690000	-0.7707620000
C	-1.4313870000	-0.3804140000	1.0006990000
H	-1.4764100000	-1.2876890000	1.6128810000
H	-1.7137630000	0.4504900000	1.6563280000
C	-2.4719070000	-0.5018060000	-0.1337400000
H	-2.1893990000	-1.3332180000	-0.7891840000
H	-3.4348980000	-0.7754840000	0.3118580000
C	-2.6398540000	0.7721670000	-0.9654440000
H	-1.7042410000	1.0580870000	-1.4524880000
H	-3.3937910000	0.6344010000	-1.7452260000
H	-2.9596900000	1.6119330000	-0.3398580000

Ground to excited state transition electric dipole moments (Au):

state	X	Y	Z	Dip. S.	Osc.
1	-0.0427	0.0953	0.0385	0.0124	0.0016

(3) neutral DMPB ground state

Cartesian coordinates

	X	Y	Z
C	0.7114740000	2.5231750000	1.2027200000
C	0.7114740000	1.1301820000	1.1994890000
C	0.7022740000	0.4074930000	0.0000000000
C	0.7114740000	1.1301820000	-1.1994890000
C	0.7114740000	2.5231750000	-1.2027200000
C	0.7078650000	3.2259660000	0.0000000000
H	0.7214090000	3.0594640000	2.1453840000
H	0.7281050000	0.5943860000	2.1428960000
H	0.7281050000	0.5943860000	-2.1428960000
H	0.7214090000	3.0594640000	-2.1453840000
H	0.7117100000	4.3101560000	0.0000000000
C	0.7033330000	-1.1063050000	0.0000000000
H	1.2577490000	-1.4588910000	-0.8768120000

H	1.2577490000	-1.4588910000	0.8768120000
C	-0.6856570000	-1.8204660000	0.0000000000
C	-0.4236320000	-3.3383540000	0.0000000000
H	0.1448270000	-3.6421560000	0.8846560000
H	-1.3649930000	-3.8960940000	0.0000000000
H	0.1448270000	-3.6421560000	-0.8846560000
C	-1.4939510000	-1.4501890000	-1.2556760000
H	-1.7150990000	-0.3809160000	-1.2910960000
H	-0.9507080000	-1.7136210000	-2.1690350000
H	-2.4461690000	-1.9893090000	-1.2690910000
C	-1.4939510000	-1.4501890000	1.2556760000
H	-0.9507080000	-1.7136210000	2.1690350000
H	-1.7150990000	-0.3809160000	1.2910960000
H	-2.4461690000	-1.9893090000	1.2690910000

Ground to excited state transition electric dipole moments (Au):

state	X	Y	Z	Dip. S.	Osc.
1	-0.0000	-0.0000	0.0773	0.0060	0.0008

(4) Cationic *anti*-PB ground state

Cartesian coordinates

	X	Y	Z
C	-0.3716980000	-2.1089470000	1.2358250000
C	-0.3716980000	-0.7397810000	1.2377360000
C	-0.3963810000	-0.0096130000	0.0000000000
C	-0.3716980000	-0.7397810000	-1.2377360000
C	-0.3716980000	-2.1089470000	-1.2358250000
C	-0.3689780000	-2.8072190000	0.0000000000
H	-0.3735460000	-2.6661870000	2.1642230000
H	-0.3734680000	-0.1859680000	2.1692200000
H	-0.3734680000	-0.1859680000	-2.1692200000
H	-0.3735460000	-2.6661870000	-2.1642230000
H	-0.3578280000	-3.8913660000	0.0000000000
C	-0.4082610000	1.4590330000	0.0000000000
H	-0.8988590000	1.8532240000	-0.8925470000
H	-0.8988590000	1.8532240000	0.8925470000
C	1.0890770000	2.0053650000	0.0000000000
H	1.6024170000	1.6142350000	0.8812830000

H	1.6024170000	1.6142350000	-0.8812830000
C	1.0912290000	3.5329410000	0.0000000000
H	0.5992330000	3.9388840000	0.8862450000
H	2.1269240000	3.8846840000	0.0000000000
H	0.5992330000	3.9388840000	-0.8862450000

Ground to excited state transition electric dipole moments (Au):

state	X	Y	Z	Dip. S.	Osc.
1	0.0000	0.0000	0.0583	0.0034	0.0001
2	0.0000	0.0000	-0.0066	0.0000	0.0000
3	-0.3906	-1.3126	0.0000	1.8754	0.1088
4	0.0905	0.1588	0.0000	0.0334	0.0022
5	-0.0000	-0.0000	-0.0194	0.0004	0.0000
6	0.0123	0.5157	-0.0000	0.2661	0.0214
7	0.0000	0.0000	0.0250	0.0006	0.0001
8	0.2260	0.3884	-0.0000	0.2019	0.0183
9	-0.0004	-0.1331	0.0000	0.0177	0.0019
10	-0.1821	-0.8765	0.0000	0.8015	0.0878

(5) Cationic *gauche*-PB ground state

Cartesian coordinates

	X	Y	Z
C	1.8438980000	1.3154070000	0.1128120000
C	0.5652600000	1.1073320000	0.5560480000
C	-0.0117410000	-0.2054120000	0.5176790000
C	0.7669310000	-1.2911530000	-0.0214150000
C	2.0456600000	-1.0801730000	-0.4626360000
C	2.5964360000	0.2245580000	-0.4029610000
H	2.2913680000	2.3008850000	0.1504560000
H	-0.0231030000	1.9246570000	0.9551240000
H	0.3256910000	-2.2804890000	-0.0563950000
H	2.6403680000	-1.8955850000	-0.8548700000
H	3.6061000000	0.3957230000	-0.7594600000
C	-1.3776730000	-0.4530810000	0.9999080000
H	-1.4244610000	-1.4264920000	1.4990050000
H	-1.7035940000	0.3263930000	1.6905570000
C	-2.4317740000	-0.5254110000	-0.1946710000
H	-2.1099120000	-1.2939640000	-0.9013880000

H	-3.3503630000	-0.8839780000	0.2753040000
C	-2.6669550000	0.8071890000	-0.8955240000
H	-1.7842720000	1.1599270000	-1.4347830000
H	-3.4713510000	0.6928050000	-1.6267940000
H	-2.9767220000	1.5845670000	-0.1921980000

Ground to excited state transition electric dipole moments (Au):

state	X	Y	Z	Dip. S.	Osc.
1	-0.0003	-0.0496	-0.0175	0.0028	0.0001
2	0.4440	0.0234	0.0460	0.1998	0.0107
3	0.7646	0.0158	0.0321	0.5859	0.0332
4	0.8186	0.0376	-0.0040	0.6715	0.0412
5	-0.2594	-0.0213	0.0290	0.0686	0.0050
6	-0.1189	-0.0134	0.0193	0.0147	0.0012
7	-0.2351	-0.1179	0.1878	0.1045	0.0086
8	-0.0912	-0.0211	0.0233	0.0093	0.0009
9	0.0963	0.0012	-0.1243	0.0247	0.0027
10	-0.6253	-0.0030	0.0150	0.3912	0.0426

(6) Cationic DMPB ground state

Cartesian coordinates

	X	Y	Z
C	0.6859020000	2.5039010000	1.2319880000
C	0.6859020000	1.1328710000	1.2341040000
C	0.7029000000	0.3993590000	0.0000000000
C	0.6859020000	1.1328710000	-1.2341040000
C	0.6859020000	2.5039010000	-1.2319880000
C	0.6811650000	3.2017990000	0.0000000000
H	0.6915720000	3.0587510000	2.1618470000
H	0.6949650000	0.5839690000	2.1677620000
H	0.6949650000	0.5839690000	-2.1677620000
H	0.6915720000	3.0587510000	-2.1618470000
H	0.6702790000	4.2859260000	0.0000000000
C	0.7623580000	-1.0580460000	0.0000000000
H	1.2582930000	-1.4331110000	-0.8967090000
H	1.2582930000	-1.4331110000	0.8967090000
C	-0.7111770000	-1.8286200000	0.0000000000
C	-0.3620260000	-3.3197280000	0.0000000000

H	0.2063090000	-3.6076850000	0.8873440000
H	-1.2908150000	-3.8992980000	0.0000000000
H	0.2063090000	-3.6076850000	-0.8873440000
C	-1.4833010000	-1.4489900000	-1.2658870000
H	-1.7683510000	-0.3945300000	-1.2796550000
H	-0.9181860000	-1.6749810000	-2.1738930000
H	-2.4050110000	-2.0367110000	-1.3061030000
C	-1.4833010000	-1.4489900000	1.2658870000
H	-0.9181860000	-1.6749810000	2.1738930000
H	-1.7683510000	-0.3945300000	1.2796550000
H	-2.4050110000	-2.0367110000	1.3061030000

Ground to excited state transition electric dipole moments (Au):

state	X	Y	Z	Dip. S.	Osc.
1	0.0000	0.0000	0.0555	0.0031	0.0001
2	0.3861	1.1008	-0.0000	1.3608	0.0668
3	0.0000	0.0000	-0.0130	0.0002	0.0000
4	0.4039	1.1177	-0.0000	1.4124	0.0757
5	-0.0000	0.0000	0.0592	0.0035	0.0002
6	-0.0000	0.0000	0.0327	0.0011	0.0001
7	-0.0524	0.0003	0.0000	0.0027	0.0002
8	0.0000	-0.0000	0.1626	0.0264	0.0022
9	0.1158	-0.3107	-0.0000	0.1099	0.0093
10	0.1202	0.3147	-0.0000	0.1135	0.0102
